# Population genetic structure and connectivity of deep‐sea stony corals (Order Scleractinia) in the New Zealand region: Implications for the conservation and management of vulnerable marine ecosystems

**DOI:** 10.1111/eva.12509

**Published:** 2017-07-20

**Authors:** Cong Zeng, Ashley A. Rowden, Malcolm R. Clark, Jonathan P. A. Gardner

**Affiliations:** ^1^ College of Animal Science and Technology Hunan Agricultural University Changsha China; ^2^ School of Biological Sciences Victoria University of Wellington Wellington New Zealand; ^3^ National Institute for Water and Atmospheric Research Kilbirnie Wellington New Zealand

**Keywords:** deep‐sea conservation, gene flow, genetic connectivity, marine protected areas, Scleractinia

## Abstract

Deep‐sea stony corals, which can be fragile, long‐lived, late to mature and habitat‐forming, are defined as vulnerable marine ecosystem indicator taxa. Under United Nations resolutions, these corals require protection from human disturbance such as fishing. To better understand the vulnerability of stony corals (*Goniocorella dumosa*,* Madrepora oculata*,* Solenosmilia variabilis*) to disturbance within the New Zealand region and to guide marine protected area design, genetic structure and connectivity were determined using microsatellite loci and DNA sequencing. Analyses compared population genetic differentiation between two biogeographic provinces, amongst three subregions (north–central–south) and amongst geomorphic features. Extensive population genetic differentiation was revealed by microsatellite variation, whilst DNA sequencing revealed very little differentiation. For *G. dumosa*, genetic differentiation existed amongst regions and geomorphic features, but not between provinces. For *M. oculata*, only a north–central–south regional structure was observed. For *S. variabilis*, genetic differentiation was observed between provinces, amongst regions and amongst geomorphic features. Populations on the Kermadec Ridge were genetically different from Chatham Rise populations for all three species. A significant isolation‐by‐depth pattern was observed for both marker types in *G. dumosa* and also in *ITS* of *M. oculata*. An isolation‐by‐distance pattern was revealed for microsatellite variation in *S. variabilis*. Medium to high levels of self‐recruitment were detected in all geomorphic populations, and rates and routes of genetic connectivity were species‐specific. These patterns of population genetic structure and connectivity at a range of spatial scales indicate that flexible spatial management approaches are required for the conservation of deep‐sea corals around New Zealand.

## 1 INTRODUCTION

The deep sea is the largest habitat on Earth, but knowledge about this biome is limited because of the logistical constraints and costs of sampling such a large and inaccessible area (Ramirez‐Llodra et al., 2011). Whilst deep‐sea communities and their ecological structures and functions are still being described, they face ongoing or increasing threats from anthropogenic activities such as fishing, mining, dumping, pollution and climate change (Ramirez‐Llodra et al., 2011). To reduce the impact of human activities in the deep sea, vulnerable marine ecosystems (VMEs) have been selected as a protection priority under United Nations General Assembly resolutions (61/105 and 59/25). VMEs are habitats and ecosystems characterized by uniqueness or rarity of their species, their significant ecological function, that are easily disturbed by anthropogenic activities and that may exhibit slow or even no recovery from disturbance (FAO, 2009). Based on the FAO characteristics, Parker, Penney, and Clark (2009) identified sponges, anemones, soft corals, sea fans, sea pens, stony corals, black corals, hydrocorals, sea lilies and armless sea stars as VME indicator taxa. The presence of these taxa can be used to identify VMEs that may be vulnerable to impacts from fishing activities in the South Pacific Ocean and guide management measures designed to protect them (Penny, Parker, & Brown, 2009).

Deep‐sea stony corals (Order Scleractinia) may form complex three‐dimensional structures that are classified as VMEs because they provide habitats and refuges for many other species (e.g., Bongiorni et al., 2010). Amongst these habitat‐forming corals, the three most common species in the New Zealand region are *Goniocorella dumosa, Madrepora oculata* and *Solenosmilia variabilis*, and these three corals are commonly found on seamount features (Tracey, Rowden, Mackay, & Compton, 2011). Seamounts (including knolls and hills) are the focus of several deep‐sea fisheries in New Zealand waters, and both *G. dumosa* and *S. variabilis* have been recorded in large quantities as bycatch from seamount fisheries (Anderson & Clark, 2003). In addition to seamounts, *G. dumosa* is also associated with phosphorite nodules on soft sediments of the Chatham Rise to the east of New Zealand (Kudrass & Rad, 1984), which suggests that *G. dumosa* may be impacted by any future mining of this resource. Unfortunately, the mode of larval development of these three corals is still poorly understood, and information about their patterns of larval movement and gene flow (connectivity) is sparse (e.g., Addamo, Reimer, Taviani, Freiwald, & Machordom, 2012; Baco et al., 2016; Miller & Gunasekera, 2017).

**Figure 1:**
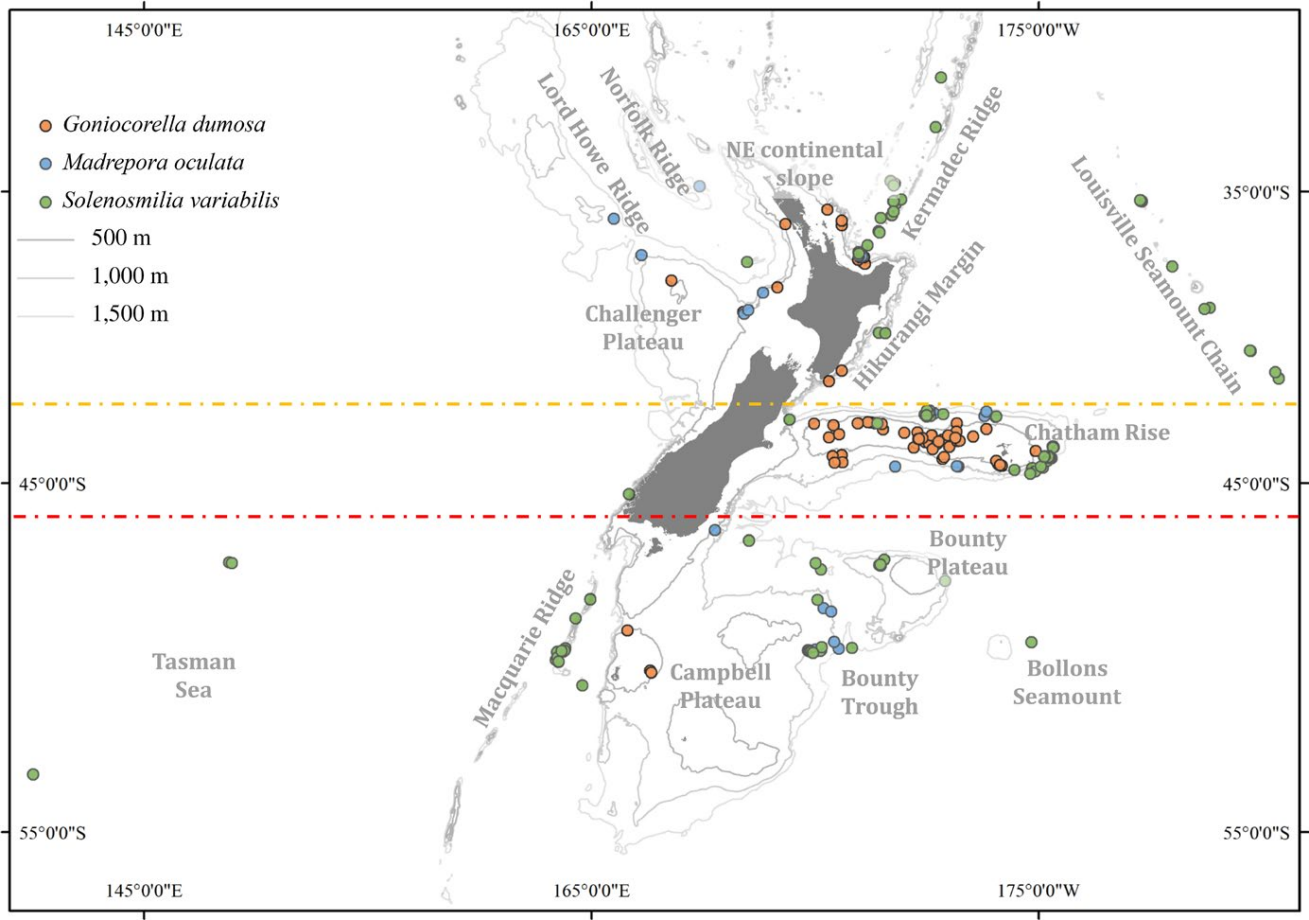
Map showing the distribution of samples amongst lower bathyal biogeographic provinces (yellow dashed line is the boundary between the northern and southern provinces), regions (yellow and red dashed lines indicate the boundaries for the north‐central‐south regions), and geomorphic features (named features) used for the analysis of genetic population structure for three species of deep‐sea stony corals

An understanding of connectivity is important for management decisions related to the conservation of corals because if limited gene exchange exists amongst populations, then loss of areas of reef may be detrimental to the overall genetic diversity of the species. In this article, we describe species‐specific patterns of genetic connectivity amongst populations of three deep‐sea corals from the New Zealand region. Newly developed microsatellite markers were genotyped, and a nuclear gene (internally transcribed spacer, *ITS*2) and a mitochondrial region (the control region, *D‐loop*) were sequenced for the three VME indicator species, *G. dumosa, M. oculata* and *S. variabilis*. We employed a province, region and geomorphic feature hierarchical framework to test the hypotheses that (i) the water mass characteristics of two biogeographic provinces (based on Watling, Guinotte, Clark, & Smith, 2013) influence the north–south population distributions, which in turn will be reflected in a province‐scale pattern of genetic structure; (ii) current flows from north and south of New Zealand that meet along the Chatham Rise to the east and that form the Subtropical Front will act as conduits and barriers to larval dispersal, which will be reflected in a north–central–south regional‐scale pattern of genetic structure, and higher genetic diversity in the central putative mixing region on the Chatham Rise; and (iii) hydrodynamic conditions (e.g., current flows, eddies, turbulent mixing) associated with particular topographic features (such as slopes, seamounts, plateaux, rises, ridges, troughs, basins) influence the dispersal of larvae, potentially restricting dispersal amongst these features, and thereby generating genetic structure amongst populations of corals found on these features (Figure 1). Tests were also conducted to examine the influence of geographic distance and depth on genetic population structure. Post hoc analyses were conducted on data to examine patterns of genetic population structure and potential barriers to gene flow independent of the hypothesis testing framework and to identify potential larval migration patterns. Assessments of effective population size were also made. Results of these analyses are interpreted in terms of their significance for management of VME indicator species around New Zealand and the possible distribution of offshore marine protected areas.

## 2 MATERIALS AND METHODS

### 2.1 Samples

The total number of specimens was 78 for *M. oculata*, 134 for *G. dumosa* and 208 for *S. variabilis*. Samples were mainly sourced from the NIWA Invertebrate Collection, which archives specimens obtained from a range of sampling expeditions since the 1950s. Samples of *G. dumosa*,* M. oculata* and *S. variabilis* were collected from depth ranges of 198–1,270 m, 236–1,537 m and 322–1,805 m, respectively. Most specimens that were used in this study were preserved in ethanol, and the rest were dry‐preserved. The majority of specimens were from seamount and slope habitats (198–1,805 m water depth) (Figure 1). Preliminary testing revealed that most specimens more than 5 years old did not provide sufficient quantity and/or quality of DNA, meaning that temporal variability amongst our samples was low, especially in the context of species that are long‐lived. Specimens within 1° of latitude and longitude were grouped for haplotypic distribution charts, and the groups were further assigned into different populations at scales that reflect the various environmental features that could influence connectivity amongst populations (i.e., province, region, geomorphic feature). Due to patchy sampling efforts and the different distributions of the species, samples for all species were not available from all locations. To achieve a balance between the validation of results and extracting maximum information content from the specimens, the minimum sample size was set at four for population analysis.

### 2.2 Molecular methods

All methods follow Zeng (2016). DNA sequencing of one mitochondrial region (*D‐loop*) and one nuclear region (*ITS*) was employed to estimate genetic connectivity and population genetic differentiation (Table S1). *ITS* data were obtained for all three species, whereas *D‐loop* data were obtained for two species (the *D‐loop* is absent in *M. oculata*). In total, 27 microsatellite loci were assayed for *G. dumosa* and *S. variabilis*, and 11 microsatellite loci were assayed for *M. oculata*. Of these loci, most were developed for this study (Zeng, 2016), but others were obtained from colleagues (Karen Miller, AIMS, Australia, for *S. variabilis* and Sophie Arnoud‐Haond, ‎IFREMER, France, for *M. oculata*).

### 2.3 Data analysis

#### 2.3.1 D‐loop and ITS variation

Multiple sequences were aligned using the plugin ClustalW Alignment (Gap Open Cost = 100, Gap Extend Cost = 10), and then all alignments were viewed by eye using Geneious v7 (Biomatters Ltd, New Zealand). For all populations with two or more individuals, the intraspecific genetic diversity was evaluated by computing the number of haplotypes, the number of polymorphic sites, haplotypic diversity (h) and nucleotide diversity (π).

Analyses of molecular variance (AMOVA) between locations (between north and south biogeographic provinces, or amongst northern, central and southern regions, or amongst geomorphic features) were tested using Arlequin (Excoffier & Lischer, 2010). Pairwise comparisons of population differentiation and significance values were estimated after 1,000 permutations. Between‐location or within‐location Φ_ST_ statistics (based on sequence divergence) were calculated to test for genetic differentiation amongst populations. If significant differentiation amongst populations was detected, the location of the genetic discontinuity was identified using BARRIER v2.2 (Manni, Guerard, & Heyer, 2004).

To visualize spatial patterns of genetic variation, specimens were colour‐coded according to haplotype and their geographic coordinates of collection were plotted using ArcGIS (ESRI, USA). The Mantel test (Mantel, 1967) was employed to test for isolation by depth by comparing the matrix of Φ_ST_ values to the matrix of depth (m) values and to test for isolation by distance by comparing the matrix of Φ_ST_ values to the matrix of shortest actual distances (km) between pairs of sites (GenAlEx v6.5, Peakall & Smouse, 2012).

#### 2.3.2 Microsatellite variation

Micro‐Checker v2.2.1 with default settings was used to identify stuttering, large allele dropout, and null alleles (van Oosterhout, Hutchinson, Wills, & Shipley, 2004). Loci that were putatively neutral or under selection were identified using LOSITAN with default settings (Antao, Lopes, Lopes, Beja‐Pereira, & Luikart, 2008). Loci under selection and loci with null alleles present at >10% frequency (Oosterhout score) were removed, and a second reduced data set (neutral loci) was created. Both the full and the reduced data sets were tested, where appropriate, for population genetic differentiation because non‐neutral loci may often be informative about population genetic structure and therefore useful for management purposes (e.g., Gagnaire et al., 2015; Wei, Wood, & Gardner, 2013). Allelic frequencies, number of alleles, departures from Hardy–Weinberg equilibrium (HWE), linkage disequilibrium (LD) and observed and expected heterozygosities were estimated using GenAlEx. Arlequin was used to estimate unbiased estimator of Wright's *F* statistic (*F*_ST_), and hierarchical *F*_ST_ analyses (AMOVA) were conducted in GenAlEx. If significant differentiation amongst populations was detected, the location of the genetic discontinuity, isolation by depth and isolation by distance were estimated as described for the *D‐loop* and *ITS* markers. Multilocus matches were employed to detect the asexual/colonial relationship between individuals (GenAlEx).

Discriminant analysis of principle components (DAPC) (Jombart, Devillard, & Balloux, 2010) was used to identify population structure amongst the microsatellite multilocus genotypes of all individuals per species. Analysis was implemented in the R package “adegenet” (Jombart, 2008). The optimal number of genetic clusters (*K*) was chosen when Bayesian information criterion (BIC) values were the lowest. Scatter plots of microsatellite genotypes in relation to discriminant functions were created in “adegenet”. STRUCTURE (v2.3.4), with a 5 × 10^6^ burn‐in period and 5 × 10^6^ MCMC runs after burn‐in, was employed to infer the number of distinct genetic groups based on Bayesian assignment analysis (Falush, Stephens, & Pritchard, 2007). The models were run in three iterations for *K* (number of distinct genetic clusters) values to evaluate likelihood scores and consistency amongst runs. The values of *K* were set from 1 to the number of geomorphic feature populations, that is, 4, 4 and 8 for *G. dumosa*,* M. oculata* and *S. variabilis*, respectively. The optimal number for *K* was chosen as the maximum number of clusters which yielded likelihoods lower than those observed at lower values of *K*. This number was computed in the online program STRUCTURE HARVESTER (Earl & von Holdt, 2012).

Where AMOVA results indicated significant genetic structure at the three different spatial scales, we used assignment tests implemented in GeneClass2 (Piry et al., 2004) to generate estimates of contemporary dispersal and to identify first‐generation migrants (e.g., Wei et al., 2013). Although this assignment methodology assumes HWE, simulations suggest that small heterozygote deficits have little effect on test performance (Cornuet, Piry, Luikart, Estoup, & Solignac, 1999). This approach does not require that the true population of origin has been sampled and it is therefore likely to be a relatively accurate method (Berry, Tocher, & Sarre, 2004).

Effective population size (*Ne*) was estimated using the software NeEstimator v2 (Do, Waples, Peel, Macbeth, & Tillett, 2014). We estimated contemporary effective population size (i.e., the *Ne* estimate applies to the time period encompassed by sampling) using multilocus data in the single‐sample bias‐corrected method based on linkage disequilibrium (Waples & Do, 2010). Both the full locus and the neutral only locus data sets were analysed. P_critical_ values were set according to the formula P_critical_ >1/2S, where S = number of individuals per population per bioprovince or region or geomorphic feature. Parametric confidence intervals were estimated, and following Waples and Do (2010) negative values of *Ne* were set to infinity.

## 3 RESULTS

There was considerable variability in the number of DNA samples obtained per species and per geomorphic feature, region and province due to our ability to extract high‐quality DNA, based on the age and state of sample preservation, as well as the spatial differences in sampling effort.

### 3.1 Population diversity and structure based on mitochondrial and nuclear DNA sequence variation

#### 3.1.1 Population genetic diversity

**Figure 2:**
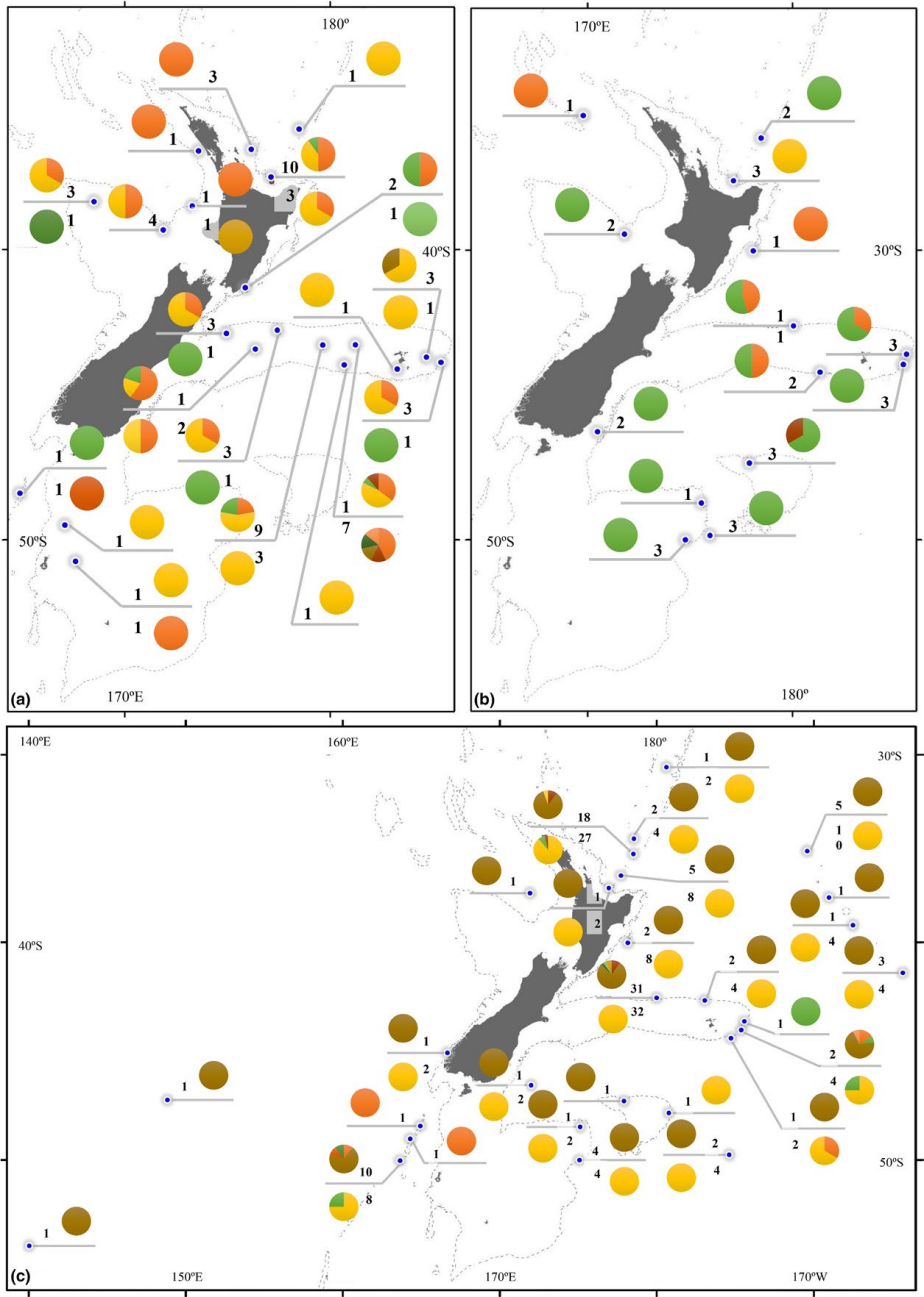
Map of haplotype distributions for *Goniocorella dumosa* (a), *Madrepora oculata* (b) and *Solenosmilia variabilis* (c) in the New Zealand region. Pie charts for *ITS* (above line) and *D‐loop* (below line) indicate haplotypic composition of each location, and numbers indicate total number of sequences from each location

For *G. dumosa*,* ITS* haplotypic diversity was greater in the southern than in the northern province, but nucleotide diversity in the northern was greater than in the southern province. *D‐loop* haplotypic and nucleotide diversity values in the southern were greater than in the northern province (Figure 2a; Table S2). However, given the small sample sizes in the south, these values must be interpreted with care. For *M. oculata*,* ITS* haplotypic and nucleotide diversity values of the northern were greater than the southern province, and values for both decreased from the north via the central to the south region (Figure 2b; Table S2). For *S. variabilis*, both the haplotypic and nucleotide diversity values of *ITS* and *D‐loop* of the southern were greater than those of the northern province, and values for both decreased from the south via the central to the north region (Figure 2c; Table S2). Common haplotypes of both *ITS* and *D‐loop* were found in most of the sampled areas for all three species (Fig. S1).

#### 3.1.2 Population genetic structure

For *G. dumosa*, AMOVA for both *ITS* and *D‐loop* did not reveal any genetic structure amongst geomorphic populations (*p* > .05 in all three cases). Comparable analyses were not possible for the between northern/southern provinces or for the amongst north‐central‐south regions because of very low sample sizes (*n* < 4) in the south. For *M. oculata*, AMOVA of *ITS* variation revealed significant differentiation amongst the three regions (north, central, south; *p* < .05). In contrast, for *S. variabilis*,* ITS* and *D‐loop* variation showed no evidence of structure at any spatial scale.

The Φ_ST_ values for *ITS* and *D‐loop* were variable but relatively low for all three species. In *G. dumosa,* no significant Φ_ST_ values were observed for either marker. For *M. oculata,* a significant pairwise value between the Kermadec Ridge and Chatham Rise populations was observed for *ITS*. For *S. variabilis,* significant pairwise population values of Φ_ST_ for *ITS* were observed between the Macquarie Ridge and Louisville Seamount Chain populations, and between the Macquarie Ridge and Kermadec Ridge populations (Table S3). No significant Φ_ST_ values were observed for *D‐loop*.

There was no statistically significant isolation by distance (*p* > .05) for any of the species, but *ITS* sequence variation exhibited a significant pattern of isolation by depth for *M. oculata* and *G. dumosa* (*p* < .05).

### 3.2 Population diversity and structure based on microsatellite variation

Micro‐Checker detected null alleles at 4, 5 and 5 loci, and LOSITAN revealed that 11, 1 and 8 loci were subject to putative selection in *G. dumosa*,* M. oculata* and *S. variabilis*, respectively. The reduced data sets (putatively neutral loci only) included eight loci for *G. dumosa*, six for *M. oculata* and 12 for *S. variabilis* (Table S4). Where appropriate, analyses were conducted on the species‐specific full (all loci) and the reduced microsatellite data sets. Because the results were concordant, we report here the results for the full data set but highlight major differences between the two data sets.

#### 3.2.1 Genotype identity test

All genotyped loci were utilized to screen out any genetically identical individuals (clones or fragments of individuals). For *S. variabilis*, one pair of specimens shared the same multilocus genotype. One specimen was excluded from analysis. In *G. dumosa*, no specimens with the same multilocus genotype were detected amongst 108 individuals. In *M. oculata*, three pairs of specimens were identical across all assayed loci, and duplicates were therefore excluded.

#### 3.2.2 Population genetic diversity

For *G. dumosa*, the number of alleles per locus varied from 7 to 27 (average = 8.20). Observed heterozygosity (*H*_O_) ranged from 0.500 to 1.000 per locus, and most *H*_O_ values were greater in all populations than expected (Table S5a). For *M. oculata*, the number of alleles per locus ranged from 5 to 19 (average = 9.96). *H*_O_ values ranged from 0.273 to 0.944 per locus, and unlike the other two coral species, were lower in all populations than expected (Table S5b). For *S. variabilis*, the number of alleles varied from 7 to 31 (average = 14.42), and *H*_O_ ranged from 0.211 to 1.000 (Table S5c). In all three species, many loci exhibited significant departures from HWE (Table S5).

Pairwise values of *F*_ST_ for all species and for both data sets were rarely greater than 0.1 (Table S6). For *G. dumosa,* only one significant result (of six tests per data set) was observed (Chatham Rise–Kermadec Ridge), no significant results were observed *M. oculata*, or for *S. variabilis,* 12 of 28 (all loci) and 14 of 28 (neutral loci) tests were significant (Table S6).

#### 3.2.3 Population genetic structure

For all three species, there was very strong concordance in AMOVA results for the reduced and for the all loci data sets, indicating that the presence or absence of a signal of genetic differentiation is conserved across all surveyed loci (Tables 1, 2, 3). The AMOVA of genetic structure for *G. dumosa* detected large‐scale structure amongst north, central, south regions and amongst the geomorphic features, but not between northern and southern provinces. The only AMOVA evidence of genetic structure in *M. oculata* was amongst the north, central and south regions. The AMOVA for *S. variabilis* detected large‐scale structure between northern and southern provinces, amongst north, central, south regions and amongst the geomorphic features. Notably, a north–central–south regional differentiation was found in all three species for both data sets (Tables 1, 2, 3).

**Table 1:** AMOVA results of microsatellites for *Goniocorella dumosa* at three different spatial scales (in bold)

Source of variation	All loci			Neutral loci only		
	*df*	ss	Var. comp.	*df*	ss	Var. comp.
**Between provinces**	1	9.373	0.000	1	3.629	0.000
Amongst Individuals	106	1,058.678	2.390**	106	422.144	0.769**
Within Individuals	108	562.500	5.208**	108	264.000	2.444**
Total	215	1,630.551	7.598	215	689.773	3.213
**Amongst regions**	2	27.947	0.088*	2	10.180	0.025*
Amongst Individuals	105	1,040.104	2.349**	105	415.593	0.757**
Within Individuals	108	562.500	5.208**	108	264.000	2.444**
Total	215	1,630.551	7.645	215	689.773	3.226
**Amongst geomorphic features**	3	0.196	0.196**	3	16.653	0.063**
Amongst Individuals	96	2.318	2.318**	96	377.757	0.740**
Within Individuals	100	5.240	5.240**	100	245.500	2.455**
Total	199	7.754	7.754	199	639.910	3.258

Significant values *p < .05, and **p < .01.

**Table 2:** AMOVA results of microsatellites for *Madrepora oculata* at three different spatial scales (in bold)

Source of variation	All loci			Neutral loci only		
	*df*	ss	Var. comp.	*df*	ss	Var. comp.
**Between provinces**	1	6.420	0.013	1	3.234	0.004
Amongst Individuals	91	507.634	1.926**	91	269.846	0.934**
Within Individuals	93	160.500	1.726**	93	102	1.097**
Total	185	674.554	3.665	185	375.081	2.035
**Amongst regions**	2	13.474	0.022*	2	7.581	0.016*
Amongst Individuals	90	500.580	1.918**	90	265.5	0.927**
Within Individuals	93	160.500	1.726**	93	102	1.097**
Total	185	674.554	3.666	185	375.081	2.039
**Amongst geomorphic features**	3	18.763	0.017	3	11.377	0.029
Amongst Individuals	55	324.898	1.886**	55	174.724	0.893**
Within Individuals	59	126.000	2.136**	59	82.000	1.390**
Total	117	469.661	4.038	117	268.102	2.312

Significant values *p < .05, and **p < .01.

**Table 3:** AMOVA results of microsatellites for *Solenosmilia variabilis* at three different spatial scales (in bold)

Source of variation	All			Neutral only		
	*df*	ss	Var. comp.	*df*	ss	Var. comp.
**Between provinces**	1	16.070	0.038*	1	11.039	0.044**
Amongst Individuals	206	2,322.236	2.207**	206	1,112.833	0.700**
Within Individuals	208	1,426.500	6.858**	208	832.500	4.002**
Total	415	3,764.805	9.103	415	1,956.373	4.747
**Amongst regions**	2	52.773	0.115**	2	32.454	0.083**
Amongst Individuals	205	2,285.532	2.145**	205	1,091.419	0.661**
Within Individuals	208	1,426.500	6.858**	208	832.500	4.002**
Total	415	3,764.805	9.119	415	1,956.373	4.746
**Amongst geomorphic features**	7	151.751	0.259**	7	80.076	0.150**
Amongst Individuals	192	2,080.067	1.971**	192	989.109	0.570**
Within Individuals	200	1,378.500	6.893**	200	802.500	4.013**
Total	399	3,610.318	9.122	399	1,871.685	4.732

Significant values *p < .05, and **p < .01.

For all three species, tests of differentiation (*F*_ST_ values) amongst populations from the geomorphic features for both the reduced and all loci data sets showed similar patterns (Table S6). In *G. dumosa*, the Kermadec Ridge population was significantly different from the Chatham Rise population (Table S6a). In *M. oculata*, no significant *F*_ST_ values were observed for pairwise testing amongst all (Campbell Plateau, Challenger Plateau, Chatham Rise and Kermadec Ridge) populations (data not shown). For *S. variabilis,* pairwise population *F*_ST_ values revealed no significant genetic differences between the Bounty Trough population and all other populations, and the Kermadec Ridge and Chatham Rise populations (Table S6b).

Tests for microsatellite genetic isolation by distance and by depth identified only two significant results—in *S. variabilis* for distance and in *G. dumosa* for depth.

### 3.3 Post hoc analyses

#### 3.3.1 Geographic differentiation

**Figure 3:**
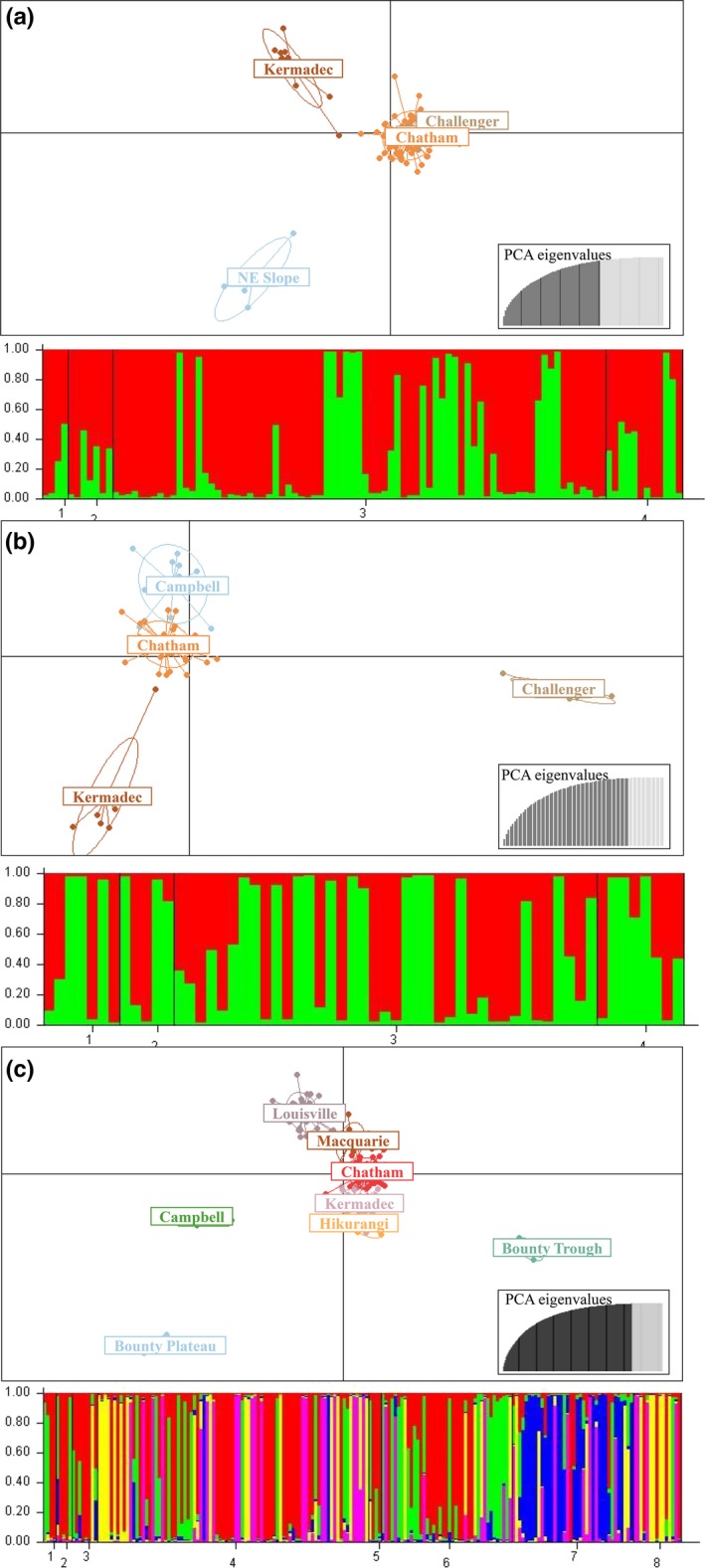
(a) DAPC scatter plot (Above) and posterior estimates from STRUCTURE (K = 2) (Below) of *Goniocorella dumosa* based on variation at all microsatellite loci. X‐axis—1. Northeast continental slope, 2. Challenger Plateau, 3. Chatham Rise, 4. Macquarie Ridge. (b) DAPC scatter plot (Above) and posterior estimates from STRUCTURE (K = 2) (Below) of *Madrepora oculata* based on variation at all microsatellite loci. X‐axis—1. Campbell Plateau, 2. Challenger Plateau, 3. Chatham Rise, 4. Kermadec Ridge. (c) DAPC scatter plot (Above) and posterior estimates from STRUCTURE (K = 5) (Below) of *Solenosmilia variabilis* based on variation at all microsatellite loci. X‐axis—1. Bounty Plateau, 2. Bounty Trough, 3. Campbell Plateau, 4. Chatham Rise, 5. Hikurangi Margin, 6. Kermadec Ridge, 7. Louisville Seamount Chain, 8. Macquarie Ridge

In *G. dumosa*, the DAPC (Figure 3a) and the STRUCTURE HARVESTER plot (Fig. S3) provided evidence of more than one population, but the number of putative genetic clusters was inconsistent between Structure (*K* = 2) and DAPC (*K* = 3, Northeast Slope, Kermadec Ridge and all other populations). In *M. oculata*, DAPC revealed that the Campbell Plateau and Chatham Rise populations were genetically similar to each other (Figure 3b). The structure complots of the posteriors from the DAPC confirm the group separations within all three species (Fig. S2), as visualized by the DAPC plots (Figure 3). The STRUCTURE HARVESTER plot indicated the presence of three groups (Fig. S3). For *S. variabilis*, the DAPC differentiated five genetic clusters, Campbell Plateau, Bounty Plateau, Bounty Trough, Louisville Seamount Chain and all other populations (Figure 3c). The best grouping cluster number was equally *K* = 3 or *K* = 5, based on Δ*K* (Fig. S3). Overall, the DAPC was able to resolve more within‐species groups than was STRUCTURE.

#### 3.3.2 Barriers to gene flow

It is likely that the BARRIER results for analysis of microsatellite variation better reflect contemporary patterns of gene flow, whereas results for DNA sequence data better reflect historical (phylogeographic) patterns of gene flow.

**Figure 4:**
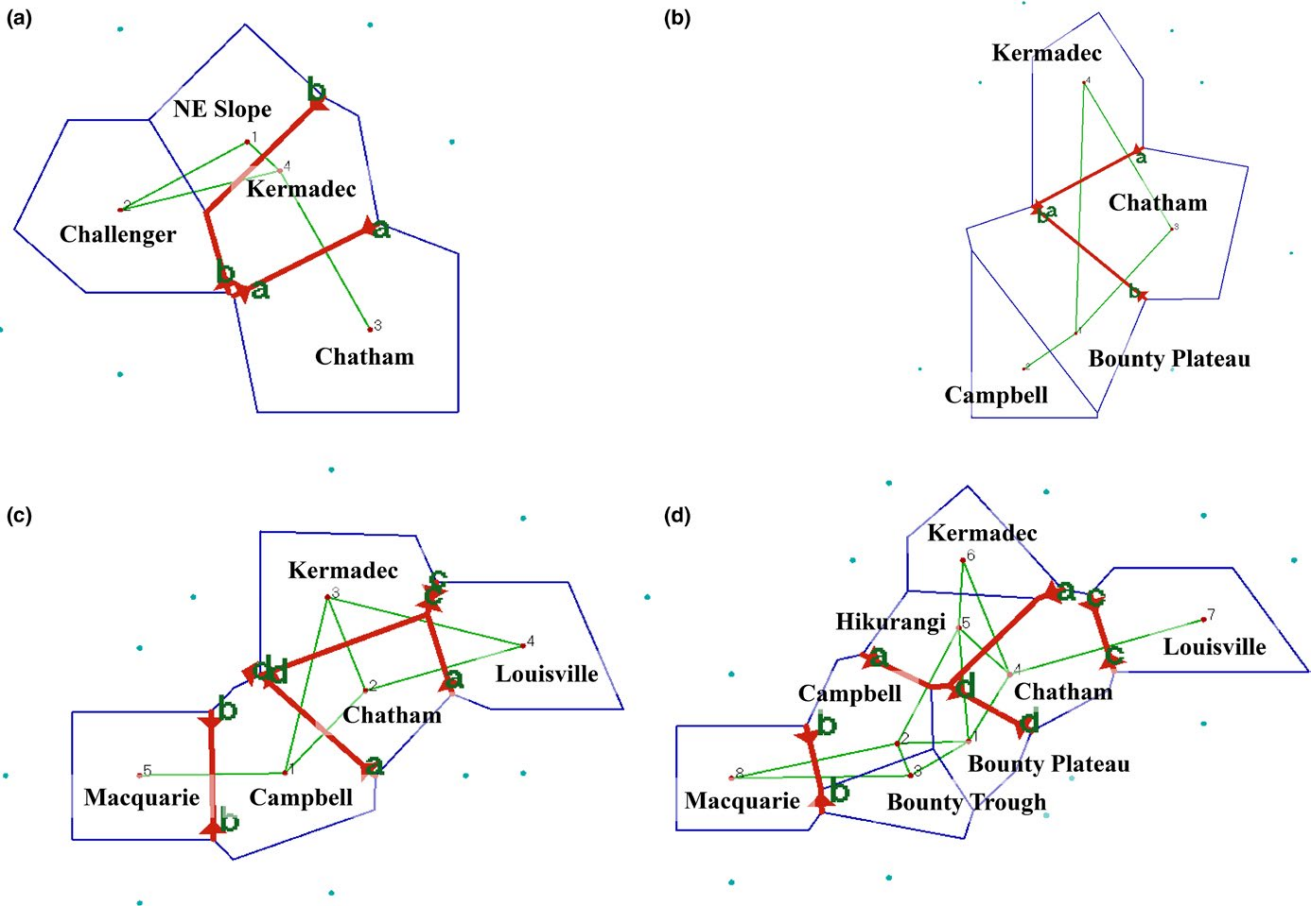
Barriers to gene flow indicated by software Barriers based on the full microsatellite data set for *Goniocorella dumosa* (a), ITS data for *Madrepora oculata* (b), ITS data for *Solenosmilia variabilis* (c) and the full microsatellite data set for Solenosmilia variabilis (d)

For *G. dumosa*, based on microsatellite variation, BARRIER predicted that barriers to gene flow may exist for the Challenger Plateau and Chatham Rise populations (Figure 4a). In *M. oculata*, because there were no significant pairwise *F*_ST_ values calculated from the microsatellite data sets, the barriers to gene flow were predicted from the Φ_ST_ values of the *ITS* sequence, which indicated that the Kermadec Ridge (to the north) and Chatham Rise populations are isolated (Figure 4b). There was generally very good agreement for the location of barriers to gene flow amongst populations of *S. variabilis* based on Φ_ST_ values of the *ITS* sequence (Figure 4c) and *F*_ST_ values of the full microsatellite data set (Figure 4d). In both instances, four barriers to gene flow were predicted, isolating the far north (Kermadec Ridge), the far east (Louisville Seamount Chain), the east (Chatham Rise), the south central (Campbell Plateau, Bounty Plateau, Bounty Trough) and the far south (Macquarie Ridge) populations.

#### 3.3.3 *Ne* estimation

Estimates of *Ne* determined from the software NeEstimator indicated that in most cases, contemporary effective population size was very small and in the range of ~20 to 60 individuals (Table S7). Estimates of *Ne* based on variation in the all loci data set were usually slightly higher than those based on the neutral loci data set. The *Ne* estimates for all three species at all three spatial hierarchies were very low.

#### 3.3.4 Assignment accuracy and estimates of first‐generation migrants

For *G. dumosa*, assignment rate success ranged between 51.9% (neutral data set, regional spatial scale) and 69.4% (all loci, regional spatial scale) (Table S8a, c). At both spatial scales, the assignment success rates were higher for all loci than for the neutral loci. First‐generation migrants were observed amongst all regions, but were more numerous for the north and central than for the south region (Table S8b). First‐generation migrants were observed between most, but not all, pairs of populations on geomorphic features (Table 8Sb). Most first‐generation migrants were associated with the Chatham Rise (Table 8Sb). The percentage of first‐generation migrants ranged from 30.6% (all loci, regional spatial scale) to 48.2% (neutral loci, regional spatial scale).

For *M. oculata*, assignment success rates at the regional scale were low (all loci = 34.2%; neutral loci = 39.5%). Individuals sampled from the central region were assigned at nearly equal rates to the north, central and south regions, whilst individuals sampled from the north or south regions exhibited higher rates of assignment success (Table S8e). First‐generation migrants were identified in all cases except one (from south to north, all loci) and occurred at high rates (65.7% of all individuals for the all loci data set, 60.5% for the neutral data set) (Table S8f).

For *S. variabilis*, assignment success rates at the bioprovince scale were high (67.8% for all loci, 63.9% for the neutral loci), but were lower at the regional scale (44.7% and 48.6%, respectively) and at the geomorphic features scale (42.0% and 42.5%, respectively) (Table S8g, i, k). Estimates of first‐generation migrants were lowest at the bioprovince scale (32.2% and 36.1%, respectively), highest at the regional scale (55.3% and 51.4%, respectively) and intermediate at the geomorphic features scale (42.0% and 42.5%, respectively). First‐generation migrants were identified between all pairs of sites for the bioprovince and the regional analyses (Table S8h, j). At the geomorphic features scale, most first‐generation migrants were associated with the Chatham Rise (Table S8l); this was also the case for the central region in the regional‐scale analysis (Table S8j).

## 4 DISCUSSION

Three habitat‐forming deep‐sea corals, *G. dumosa*,* M. oculata* and *S. variabilis*, were examined for genetic connectivity and gene flow amongst populations in the New Zealand region for the purpose of contributing to effective spatial management planning options to protect VMEs. We employed an a priori hierarchical and spatially explicit approach to hypothesis testing for population genetic structure, estimated contemporary and historical effective population sizes and identified barriers to gene flow as well as source and sink populations, all of which may contribute significantly to management decisions with conservation outcomes in the deep sea.

### 4.1 Influence of sample sizes and marker selection on detection of population genetic structure

Small sample sizes and an absence of fine spatial scale sampling are widely acknowledged as inevitable constraints in genetic studies of deep‐sea organisms (reviewed by Baco et al., 2016). Although limited sampling made it difficult to reveal small‐scale population genetic structure, we develop here an approach that provides a broad spatial‐scale view of the connectivity of three coral species and that reveals sufficient information about patterns of genetic structure to be useful to support management decisions.

Preliminary data screening identified a number of outlier loci within the microsatellite data set, a well‐known problem for taxa such as deep‐sea corals. It is unknown for sure why outlier loci occur at such high frequencies, but it may be for several different reasons, including null alleles, coloniality, inbreeding, selection, the Wahlund effect and poor state of sample preservation (Baco et al., 2016; Becheler et al., 2015; Miller & Gunasekera, 2017; Quattrini, Baums, Shank, Morrison, & Cordes, 2015). We always tested our DNA to ensure that we worked with the best quality possible, so poor quality DNA (e.g., arising from age of sample or preservation in formalin) is unlikely to be an explanation. To address this problem, our analytical approach has been to test both the full locus and the neutral locus data sets, given that both may be informative. This is particularly important from the perspective of including non‐neutral loci in analyses that are (usually) designed to meet neutral expectations. Importantly, there was very little difference in the results of all analyses between the full (all loci) and the neutral (reduced number of loci) data sets. This strongly suggests that the presence/absence of a signal of genetic differentiation is conserved across all surveyed loci. That is, addition or removal of the outlier loci is not critical to the interpretation of the results in the context of management options for future protection of New Zealand's vulnerable marine ecosystems and their associated taxa.

As our hierarchical spatial testing increased in definition, so our sample sizes decreased. Thus, our analyses have small sample sizes for most geomorphic features but larger sample sizes for the regional and biogeographic province levels of testing. All estimates for populations on geomorphic features should be interpreted with care and are best viewed in combination with other analyses. However, population groups (provinces and regions) and populations on some geomorphic features (such as the Chatham Rise) had large enough sample sizes to reveal patterns of genetic differentiation and connectivity. Overall, our analyses are probably best viewed as a conservative interpretation of patterns of population genetic differentiation.

Use of the software package STRUCTURE (Falush et al., 2007) to identify the number of distinct genetics groups (i.e., identification of *K*) is now widespread in population genetics analyses (e.g., Ruiz‐Ramos, Saunders, Fisher, & Baums, 2015). STRUCTURE is a parametric analysis and is known to perform best when sample sizes are equal, when null alleles are absent and when populations are in HWE (Puechmaille, 2016; Putman & Carbone, 2014; Wang, 2017). Our analyses using STRUCTURE with no a priori groupings did not provide reliable results, most probably because of large differences in population/region sample sizes and the absence of HWE in several samples. Absence of HWE amongst deep‐sea corals, in particular the presence of significant heterozygote deficiencies, is routinely reported (Baco, Clark, & Shank, 2006; Le Goff & Rogers, 2002; Lunden, McNicholl, Sears, Morrison, & Cordes, 2014; Miller & Gunasekera, 2017). One interpretation of multilocus heterozygote deficiencies is that recruitment in many populations is locally derived with infrequent long‐distance dispersal events. Such an interpretation is consistent with our results for the three coral species we examined (see below). Because of the uncertainty associated with the STRUCTURE results, we have focussed on other analyses for which there are greater robustness and more confirmation from other approaches.

Miller, Williams, Rowden, Knowles, and Dunshea (2010) noted that low genetic diversity of nuclear and mitochondrial DNA sequences limits the utility of these markers in population genetics studies of *S. variabilis* and *M. oculata*. In the present study, genetic diversities of *ITS* and *D‐loop* regions were low, and neither marker provided evidence of population structure in *G. dumosa* or *S. variabilis*, but *ITS* in *M. oculata* revealed a north‐central‐south regional pattern of differentiation. There were no unique haplotypes in any of the geomorphic populations and only four significant Φ_ST_ values were observed between pairs of populations on geomorphic features (Fig. S1). These findings for the DNA sequences indicate that insufficient variation exists for these markers types to be informative at the scale of our work. In contrast, microsatellite variation provided much greater resolution of genetic structure, connectivity and isolation. Where significant results exist for both marker types for the same species, they are in agreement (e.g., location of putative barriers to gene flow and estimation of exchange of migrants). In the present study, to obtain a comprehensive understanding of the population genetic structure of the three corals, both DNA sequence and microsatellite marker types are considered, and robust results are highlighted when both marker results are in agreement.

### 4.2 Effective population sizes

Estimation of effective population size (*Ne*) depends on a signal that is a function of 1/*Ne*. Such methods are therefore most powerful with small populations when the signal is strong, but have difficulty distinguishing large populations from infinite ones when the signal is small (Waples & Do, 2010). Our estimates of *Ne* were typically in the range of 20–60 individuals, meaning that at all spatial scales all three corals have very small effective contemporary population sizes (Waples & Do, 2010). This may occur because these species are typically late (old) to reach sexual maturity with the result that few adult (mature) colonies exist in an area and therefore few colonies contribute to reproductive success. Alternatively, given the topographic complexity of the seafloor habitat in general, it is possible that certain locations are favoured in terms of reproductive success for corals because of local topography and/or its interaction with local currents. Thus, whichever individual is in possession of a “good” spot may be able to contribute disproportionally more to future generations that any number of other individuals that occupy poorer quality locations. From a management or protection perspective what is of considerable concern here is that all species show such small effective population sizes. This result is consistent with recently published estimates of low *Ne* values for *S. variabilis* from the Australian EEZ (Miller & Gunasekera, 2017). This implies that any (further) damage to the corals could have a serious negative effect on the regional population.

Historical events will likely have altered the ancestral and contemporary effective sizes of populations (Palstra, O'Connell, & Ruzzante, 2007). Trying to quantify ancestral values of *Ne* is problematical, in particular for deep‐sea taxa when sample sizes are small and spatial coverage is limited in its extent (Baco et al., 2016). The force altering contemporary *Ne* values is expected to be destruction from fishing activity, given that no other impact is known to have such a potentially broad spatial effect in the region. However, the extent of damage to corals caused by bottom trawling is not quantified, although qualitative estimates indicate that such damage can be extensive, in particular in localized areas around fishing hotspots, such as the seamounts where corals are predominantly found (Althaus et al., 2009; Clark & Rowden, 2009; Parker et al., 2009). Other factors, including environmental factors such as depth‐dependent water temperature, *p*H, calcite and aragonite saturation concentrations, are also likely to contribute both historically and contemporaneously (Henry et al., 2014; Miller, Rowden, Williams, & Häussermann, 2011; Tracey et al., 2013), but are thought to be of lesser importance than fishing pressure in modern times. However, the most recent study of possible impacts of fishing activity on deep‐sea corals (Miller & Gunasekera, 2017) reported that genetic diversity of *S. variabilis* and the solitary cup coral *Desmophyllum dianthus* on fished and unfished seamounts was similar. This question clearly requires further investigation.

### 4.3 Patterns of genetic connectivity

Populations of *M. oculata* exhibited regional structure, populations of *G. dumosa* were differentiated at the regional and geomorphic features scale, and populations of *S. variabilis* exhibited structure between provinces, amongst regions and also amongst geomorphic features. In other comparable studies, little or no evidence of province‐scale structure has been observed amongst populations of crustaceans and sponges in the New Zealand region (Bors, Rowden, Maas, Clark, & Shank, 2012; Zeng, 2016). Thus, we conclude that there is little support for the hypothesis that water mass characteristics associated with biogeographic provinces within the New Zealand region result in genetic population structure of *G. dumosa* and *M. oculata*. However, a north‐central‐south regional pattern of structure was observed in another deep‐sea scleractinian, the cup coral, *D. dianthus* based on *ITS* variation (Miller et al., 2010), and also in the deep‐sea sponge *Poecillastra laminaris* based on *COI*,* Cytb* and microsatellite variation (Zeng, 2016). In combination, these findings provide evidence for the hypothesis that currents/fronts associated with the Chatham Rise act as a barrier to gene flow for several species across multiple phyla. Significant genetic differentiation has already been reported at the geomorphic population level in noncoral species between, for example, the Challenger Plateau and the Chatham Rise (Bors et al., 2012; Knox et al., 2012). These results, combined with those for the deep‐sea corals, support the hypothesis that oceanic dynamics contribute to the formation of fine‐scale population structure amongst populations on different geomorphic features in the New Zealand region.

### 4.4 Oceanographic dynamics

We hypothesized that currents associated with the Subtropical Front would influence north‐central‐south regional scale population connectivity, which was indeed observed for all three corals. Surface and intermediate currents flow from northwest New Zealand in a southerly direction to the Chatham Rise following the eastern coastline of the North Island. Currents also flow from the southeast of New Zealand northward along the east coast of the South Island towards the Chatham Rise. The result is that currents from the warmer north and the cooler south meet and mix along the Chatham Rise, forming the Subtropical Front, before heading east into the Pacific Ocean (Chiswell, Bostock, Sutton, & Williams, 2015). These two contrasting sets of currents, their associated eddies and the Subtropical Front may therefore impede larval migration between the south and north regions and provide an explanation for the increased genetic diversity values observed amongst populations on the Chatham Rise (central region) for all three corals and also for some sponges (Zeng, 2016).

At the geomorphic features level, our analyses provided specific evidence for a lack of genetic connectivity between populations on the Kermadec Ridge and the Chatham Rise (significant *F*_*ST*_ and clear split in DAPC plots). No previous study has investigated connectivity between these two areas, but significant genetic subdivision between populations of *D. dianthus* was detected between the Auckland Island slope (far south) and seamounts on the northern Chatham Rise (Miller et al., 2010). This differentiation is consistent with patterns of flow at depth, with water from the far south flowing north until it reaches the southern edge of the Chatham Rise, before it is deflected eastward (Chiswell et al., 2015). In other words, migrants from the Auckland Island slope are not expected to reach the northern edge of the Chatham Rise, which will result in some degree of genetic differentiation, which is indeed what is observed. For *S. variabilis*, the most apparent difference between populations on geomorphic features was that between the Louisville Seamount Chain and all other populations. The general lack of current flow from the Louisville Seamount Chain towards most topographic features elsewhere in the New Zealand region (Chiswell & Rickard, 2006) is likely to be the reason that populations of *S. variabilis* on this feature are differentiated genetically from those in the rest of the region.

### 4.5 Depth and related environmental gradients

Depth is an important factor contributing to patterns of genetic connectivity for some benthic fauna, particularly for taxa that are distributed across a wide depth range (Brandão, Sauer, & Schön, 2010; Miller et al., 2010; O'Hara, England, Gunasekera, & Naughton, 2014; Ruiz‐Ramos et al., 2015). In the present study, there was no evidence for isolation by depth (across the depth range 322–1,805 m) for *S. variabilis*. Populations of *S. variabilis* from Australian waters were also not differentiated by depth, but populations were sampled within a more restricted depth range (1,000–1,400 m) (Miller & Gunasekera, 2017). For populations of both *G. dumosa* (from 300 to 600 m) and *M. oculata* (from 700 to 1,200 m), at least one marker revealed the existence of a pattern of isolation by depth. As neither *G. dumosa* nor *M. oculata* populations were genetically isolated by distance, this implies that depth and related environmental gradients may play an important role in the larval migration of these species. Within the New Zealand region, Miller et al. (2011) reported that the stony cup coral *D. dianthus* from different depth strata (<600 m, 1,000–1,500 m, >1,500 m) are strongly differentiated based on DNA sequence variation. Unfortunately, the limited sample sizes of the present study precluded detailed analysis to determine at what depth(s) genetic differentiation for *G. dumosa* and *M. oculata* populations takes place (i.e., the response may just as easily be a gradient as an abrupt break).

### 4.6 Life history and dispersal strategies

Differences in reproductive strategies may contribute to differences in patterns of gene flow and genetic structure observed across taxa in the deep sea (Hilário et al., 2015). All three species showed evidence of self‐recruitment (i.e., high self assignment rates) at larger spatial scales which doubtless have contributed to the patterns of genetic structure detected in all three species, as well as the limited connectivity that exists amongst regions. Interestingly, the migration estimates amongst populations on different geomorphic features for the three corals may be related to maximum oocyte size. *M. oculata* has the largest mean oocyte size (2~3 times larger than other two species) (Table S9) and was the only species for which significant differentiation amongst populations on geomorphic features was not observed (it also had the lowest assignment success rates, something that is consistent with higher levels of gene flow), whereas *G. dumosa* and *S. variabilis* with a smaller mean oocyte diameter exhibited less connectivity (i.e., significant structure existed amongst populations on geomorphic features and rates of assignment success were lower). Given its similarity to *S. variabilis* in terms of morphology and reproduction, the larvae of *G. dumosa* are likely to share the same characteristics. Based on this assumption, the larger oocyte size, providing more nutritional support, may promote a longer larval life and greater dispersal potential for *M. oculata*. Thus, life‐history characteristics may vary even between closely related deep‐sea corals (Burgess & Babcock, 2005) and therefore can influence patterns of population connectivity, as recently reported by Miller and Gunasekera (2017) for *S. variabilis* (limited dispersal) and *D. dianthus* (widespread dispersal).

In the present study, none of the three coral species showed strong evidence of asexual reproduction as assessed using multilocus microsatellite genotypes. Whilst there was no direct evidence to indicate asexual reproduction in any of the three species, the smaller effective population size with heterozygote deficits at most loci of *S. variabilis* suggests that this species has a higher asexual or inbreeding rate in populations on geomorphic features than the other two species. Miller and Gunasekera (2017) predicted that clonal reproduction may account for as much as 76% of recruitment in Australian populations of *S. variabilis*, and if applicable in the New Zealand context, then this high asexual recruitment rate would explain the self‐recruitment result in this study. Asexual reproduction has also been observed in *G. dumosa*, but the proportion of clonal reproduction in recruitment is unknown, whilst for *M. oculata,* there is no evidence for asexual reproduction (Burgess & Babcock, 2005). However, it has been reported that mixed clonal/sexual reproduction is nearly indistinguishable from strict sexual reproduction as long as the proportion of clonal reproduction is not large (Balloux, Lehmann, & De Meeûs, 2003). Therefore, asexual reproduction would not be the main explanation for the genetic structure and connectivity patterns of three corals observed in this study.

### 4.7 Conservation and management implications

Seventeen seamount closure areas and 17 Benthic Protection Areas (BPAs) were established in 2001 and 2007, respectively, to protect benthic fauna (including VMEs such as coral reefs) from bottom trawl fisheries throughout the New Zealand EEZ (Fig. S4). These closed areas were not designed with input from population connectivity studies beyond the general principle that protected areas should be large and distributed amongst different environments throughout the EEZ. Understanding connectivity amongst areas is fundamental to designing an effective and new protected areas network or for modifying the existing distribution of protected areas to create a network (Hilário et al., 2015; Palumbi, 2003). The maintenance of genetic diversity and the protection of genetically distinct populations should also be considered as an integral component of protected area design (Bors et al., 2012).

The results of the present study for *G. dumosa, M. oculata* and *S. variabilis* (as proxies for similar VME indicator species) provide new information useful for assessing the effectiveness of existing protection measures, and the design of future protected area networks in the EEZ. The populations of the three species all showed evidence of pronounced self‐recruitment within the scale of the geomorphic features examined. This finding suggests that additional protected areas will be required to maintain genetic diversity (through self‐recruitment) of populations at features that do not already receive some protection. For *S. variabilis*, populations on all the geomorphic features examined were to some extent source populations for other populations, but those from the Kermadec Ridge and the Bounty Trough seem to be particularly important. Of particular significance as a migrant source for *G. dumosa* and *S. variabilis* populations was the Kermadec Ridge, with the NE Slope (*G. dumosa*), the Bounty Trough (*S. variabilis*) and Louisville Seamount Chain (*S. variabilis*) populations also being important for genetic connectivity.

Currently, most geomorphic features examined contain one or more BPAs or seamount closure areas. The exceptions are the Hikurangi Margin, Bounty Trough and Macquarie Ridge, as well as the Lord Howe Rise and the Louisville Seamount Chain which are outside the New Zealand EEZ. The Hikurangi Margin is an area subject to bottom trawling (Clark & O'Driscoll, 2003), and populations of VME indicator taxa, such as reef‐forming corals, are therefore at risk from disturbance. The Bounty Trough is a geomorphic feature where deep‐water drilling for hydrocarbons may occur in the future (Wood & Davy, 2008), making populations of VME indicator taxa that exist there also at risk from anthropogenic activities. Although there are no BPAs on the Macquarie Ridge, two seamount closures to the west and east of the ridge, and a BPA to the south, may provide some protection for this southern source of genetic connectivity. However, this will depend on whether they provide suitable habitat for the corals. Source populations of corals on the Kermadec Ridge are mainly protected from bottom trawling by the Kermadec and Tectonic Reach BPAs and will likely receive additional protection should a proposed Kermadec Ocean Sanctuary be established in the near future (http://www.mfe.govt.nz/marine/kermadec-ocean-sanctuary). However, outside of the sanctuary area corals and other VME taxa are still vulnerable to disturbance from future seabed mining of sulphide deposits in the region (Boschen et al., 2016). While there are two BPAs on the Chatham Rise, they do not cover the depth range of the two deeper‐distributed species, in particular *S. variabilis*, nor do they provide much protection along the axis of the Rise, including some areas where *G. dumosa* forms dense thickets in an area of interest for phosphorite nodule mining. Considering the relative importance of the Chatham Rise as a sink population and as a hot spot area with the highest genetic diversity but relative lower effective population sizes, additional protected areas should be considered here for the future management of human activities on this feature.

The results of the present study, and those of previous genetic connectivity studies for various species around New Zealand (Bors et al., 2012; Boschen, Rowden, Clark, & Gardner, 2015; Dueñas et al., 2016; Knox et al., 2012; Miller et al., 2010) provide genetic connectivity information at different spatial scales. Not surprisingly, their connectivity patterns vary across the range of taxa with differing ecological characteristics. These variable patterns demonstrate the need for a flexible spatial management system that can be periodically adjusted to accommodate increased understanding about the connectivity of a range of deep‐sea benthic taxa at a variety of spatial scales.

## DATA ARCHIVING STATEMENT

Data for this study are available from the Dryad Digital Repository: https://doi.org/10.5061/dryad.61622. *ITS* and *D‐loop* DNA sequences were also deposited in GenBank under accession numbers: MF360747–MF360784.
