# Supplementary material for: Population genetic structure and connectivity of deep‐sea stony corals (Order Scleractinia) in the New Zealand region: Implications for the conservation and management of vulnerable marine ecosystems
Source: Evol Appl. 2017 Jul 20;10(10):1040–54. doi: 10.1111/eva.12509 (PMC5680633; doi:10.1111/eva.12509)
Supplement: Supplementary file 1 [file EVA-10-1040-s001.docx]

Table S1. Details of PCR primers and conditions used to amplify two DNA regions.

| DNA region | Primer name | Primer 5’-3’ | Annealing temperature (°C) | Trimmed size (bp) |
| --- | --- | --- | --- | --- |
| *ITS* | 348 F | TTGACGGTGGATCTCTTGGC | 50 | ~400 |
|  | 854 R | CAAATTTGAGCTGGTCCCGC |  |  |
| *D-loop* | 74F | CCCAGGGGCCTTGTTCAATTTCTA | 50 | ~400 |
|  | 834R | TGGAGGGAGAGGGCAAATTCACTA |  |  |

Table S2. ITS and D-loop diversity statistics for Goniocorella dumosa, Madrepora oculata and Solenosmilia variabilis.

| Species | *Goniocorella dumosa* | | | | | | | | | | | *Madrepora oculata* | | | | | | *Solenosmilia variabilis* | | | | | | | | | | |
| --- | --- | --- | --- | --- | --- | --- | --- | --- | --- | --- | --- | --- | --- | --- | --- | --- | --- | --- | --- | --- | --- | --- | --- | --- | --- | --- | --- | --- |
| Marker | N | *ITS* | | | | | *D-loop* | | | | | N | *ITS* | | | | | N | *ITS* | | | | | *D-loop* | | | | |
| Population |  | n | s | h | Hd | π | n | s | h | Hd | π |  | n | s | h | Hd | π |  | n | s | h | Hd | π | n | s | h | Hd | π |
| Northern province | 125 | 80 | 22 | 5 | 0.636 | 0.023 | 26 | 51 | 11 | 0.825 | 0.028 | 58 | 26 | 78 | 3 | 0.557 | 0.122 | 148 | 96 | 155 | 9 | 0.287 | 0.023 | 119 | 4 | 4 | 0.098 | 0 |
| Southern province | 8 | 3 | 1 | 2 | 0.667 | 0.002 | 2 | 25 | 2 | 1 | 0.048 | 16 | 10 | 1 | 2 | 0.2 | 0.001 | 36 | 25 | 149 | 5 | 0.42 | 0.055 | 22 | 2 | 2 | 0.173 | 0.001 |
| North region | 31 | 25 | 21 | 4 | 0.6 | 0.021 | 6 | 30 | 5 | 0.933 | 0.028 | 15 | 8 | 78 | 3 | 0.679 | 0.177 | 56 | 48 | 2 | 3 | 0.121 | 0 | 75 | 3 | 3 | 0.079 | 0 |
| Central region | 94 | 55 | 22 | 5 | 0.64 | 0.023 | 20 | 49 | 8 | 0.805 | 0.029 | 43 | 16 | 41 | 2 | 0.458 | 0.082 | 77 | 48 | 155 | 8 | 0.432 | 0.045 | 44 | 3 | 3 | 0.132 | 0.001 |
| South region | 8 | 3 | 1 | 2 | 0.667 | 0.002 | 2 | 25 | 2 | 1 | 0.048 | 16 | 10 | 1 | 2 | 0.2 | 0.001 | 36 | 25 | 149 | 5 | 0.42 | 0.055 | 22 | 2 | 2 | 0.173 | 0.001 |
| Kermadec Ridge | 14 | 11 | 21 | 4 | 0.709 | 0.026 | 3 | 22 | 2 | 0.667 | 0.028 | 7 | 5 | 64 | 2 | 0.6 | 0.168 | 46 | 32 | 2 | 3 | 0.179 | 0.001 | 49 | 3 | 3 | 0.119 | 0 |
| NE continental slope | 7 | 3 | 0 | 1 | 0 | 0 | - | - | - | - | - | - | - | - | - | - | - | - | - | - | - | - | - | - | - | - | - | - |
| Challenger Plateau | 8 | 8 | 12 | 2 | 0.571 | 0.021 | 2 | 12 | 2 | 1 | 0.023 | 5 | 2 | 0 | 1 | 0 | 0 | 1 | 1 | 0 | 1 | 0 | 0 | - | - | - | - | - |
| Norfolk Ridge | - | - | - | - | - | - | - | - | - | - | - | 2 | 1 | 0 | 1 | 0 | 0 | - | - | - | - | - | - | - | - | - | - | - |
| Hikurangi Margin | 2 | 2 | 13 | 2 | 1 | 0.04 | 1 | 0 | 1 | 0 | 0 | 1 | 1 | 0 | 0 | 0 | 0 | 9 | 2 | 0 | 1 | 0 | 0 | 8 | 0 | 1 | 0 | 0 |
| Chatham Rise | 94 | 55 | 22 | 5 | 0.64 | 0.023 | 20 | 49 | 8 | 0.805 | 0.029 | 43 | 16 | 41 | 2 | 0.458 | 0.082 | 77 | 48 | 155 | 8 | 0.432 | 0.045 | 44 | 3 | 3 | 0.132 | 0.001 |
| Campbell Plateau | 6 | 2 | 0 | 1 | 0 | 0 | - | - | - | - | - | 9 | 4 | 0 | 1 | 0 | 0 | 13 | 5 | 0 | 1 | 0 | 0 | 6 | 0 | 1 | 0 | 0 |
| Bollons Seamount | - | - | - | - | - | - | - | - | - | - | - | - | - | - | - | - | - | 2 | 2 | 0 | 1 | 0 | 0 | 4 | 0 | 1 | 0 | 0 |
| Bounty Plateau | - | - | - | - | - | - | - | - | - | - | - | 5 | 4 | 1 | 2 | 5 | 0.002 | 6 | 2 | 18 | 2 | 1 | 0.05 | - | - | - | - | - |
| Bounty Trough | - | - | - | - | - | - | - | - | - | - | - | 2 | 3 | 0 | 1 | 0 | 0 | 6 | 1 | 0 | 1 | 0 | 0 | 2 | 0 | 1 | 0 | 0 |
| Macquarie Ridge | 2 | 1 | 0 | 1 | 0 | 0 | 1 | 0 | 1 | 0 | 0 | - | - | - | - | - | - | 19 | 12 | 147 | 4 | 0.636 | 0.103 | 8 | 2 | 2 | 0.429 | 0.002 |
| Tasman Basin | - | - | - | - | - | - | - | - | - | - | - | - | - | - | - | - | - | 3 | 2 | 0 | 1 | 0 | 0 | - | - | - | - | - |
| Louisville Seamount Chain | - | - | - | - | - | - | - | - | - | - | - | - | - | - | - | - | - | 15 | 13 | 0 | 1 | 0 | 0 | 18 | 0 | 1 | 0 | 0 |
| Total | 133 | 83 | 22 | 6 | 0.629 | 0.022 | 70 | 51 | 12 | 0.788 | 0.027 | 74 | 34 | 78 | 4 | 0.476 | 0.095 | 186 | 104 | 156 | 11 | 0.299 | 0.031 | 139 | 3 | 3 | 0.097 | 0 |

N is the total number of individuals sampled for a site, n is the number of sequences for analysis from the site, s is the number of polymorphic nucleotide sites in the sequence, h is the number of haplotypes represented at the site, Hd is haplotype diversity, and π is nucleotide diversity.

Haplotype diversity (Hd) and nucleotide diversity (π) are expressed as mean ± SD.

Table S3. Pairwise Φ_ST_ values for ITS of Madrepora oculata (above diagonal) and Solenosmilia variabilis (below diagonal) amongst geomorphic populations

|  | Bounty Plateau | Campbell Plateau | Chatham Rise | Kermadec Ridge | Louisville Seamount Chain | Macquarie Ridge |
| --- | --- | --- | --- | --- | --- | --- |
| Bounty Plateau |  | 0.063 | 0.148 | 0.447 | - | - |
| Campbell Plateau | - |  | 0.175 | 0.5 | - | - |
| Chatham Rise | - | -0.087 |  | 0.457** | - | - |
| Kermadec Ridge | - | -0.094 | 0.019 |  | - | - |
| Louisville Seamount Chain | - | 0 | -0.014 | -0.018 |  | - |
| Macquarie Ridge | - | -0.018 | 0.032 | 0.223* | 0.100* |  |

Significant values of p < 0.05 are marked as *, and p < 0.01 are marked as **.

Data for *G. dumosa* not included because all *Φ*_ST_ values are NS.

Table S4. Locus by locus information for three species of deep-sea corals from the New Zealand region.

Table S4a. - Goniocorella dumosa

| Locus | Success | *F_IS_* | *F_ST_* | Oosterhout | Selection |
| --- | --- | --- | --- | --- | --- |
| B106 | 100.00% | -0.241 | 0.335 | -0.0278 | Neutral |
| B1 | 100.00% | -0.794 | 0.031 | -0.4978 | Balancing |
| C107 | 91.67% | 0.088 | 0.222 | 0.1480 | Neutral |
| D5 | 87.96% | -0.066 | 0.203 | 0.0730 | Neutral |
| B123 | 81.48% | -0.474 | 0.148 | 0.0130 | Balancing |
| D213 | 77.78% | -0.297 | 0.265 | -0.0046 | Neutral |
| SVA13 | 70.37% | -0.419 | 0.331 | -0.0807 | Neutral |
| C4 | 72.22% | -0.235 | 0.273 | 0.1009 | Positive |
| SVA15 | 66.67% | -0.089 | 0.258 | 0.1327 | Neutral |
| SVA10 | 60.19% | -0.172 | 0.482 | 0.0562 | Balancing |
| SVA2 | 62.96% | -0.524 | 0.208 | 0.0314 | Balancing |
| SVA18 | 60.19% | -0.276 | 0.434 | 0.0952 | Positive |
| SVA8 | 56.48% | -0.417 | 0.364 | 0.0166 | Neutral |
| D103 | 61.11% | -0.229 | 0.401 | 0.0737 | Neutral |
| SVA1 | 52.78% | 0.046 | 0.629 | 0.2778 | Positive |
| B116 | 59.26% | -0.156 | 0.352 | 0.0917 | Balancing |
| SVA11 | 52.78% | -0.853 | 0.459 | -0.4397 | Balancing |
| SVA4 | 43.52% | -0.430 | 0.553 | -0.0002 | Balancing |
| SVA7 | 36.11% | 0.185 | 0.602 | 0.1387 | Positive |
| SVA20 | 57.41% | -0.358 | 0.464 | 0.0137 | Neutral |

Table S4b. - Madrepora oculata

| Locus | Success | *F_IS_* | *F_ST_* | Oosterhout | Selection |
| --- | --- | --- | --- | --- | --- |
| c016 | 65.59% | -0.445 | 0.247 | 0.0018 | Neutral |
| g028 | 47.31% | -0.392 | 0.460 | 0.0630 | Neutral |
| c7 | 27.96% | -0.539 | 0.604 | 0.0515 | Neutral |
| g006 | 55.91% | -0.423 | 0.417 | 0.0361 | Neutral |
| g004 | 64.52% | -0.182 | 0.358 | 0.1405 | Neutral |
| g25 | 24.73% | 0.572 | 0.850 | 0.3126 | Positive |
| c013 | 73.12% | -0.319 | 0.463 | 0.1130 | Neutral |
| g016 | 30.11% | -0.263 | 0.648 | 0.0718 | Neutral |
| g022 | 30.11% | -0.044 | 0.678 | 0.2185 | Neutral |
| a5 | 55.91% | 0.009 | 0.434 | 0.2003 | Neutral |
| g010 | 61.29% | -0.333 | 0.311 | 0.1030 | Neutral |

Table S4c. - Solenosmilia variabilis

| Locus | Success | *F_IS_* | *F_ST_* | Oosterhout | Selection |
| --- | --- | --- | --- | --- | --- |
| B106 | 96.63% | -0.559 | 0.188 | -0.3679 | Neutral |
| C107 | 94.23% | -0.330 | 0.180 | -0.0057 | Neutral |
| D5 | 95.19% | -0.127 | 0.221 | 0.0257 | Neutral |
| B1 | 96.63% | -0.259 | 0.178 | -0.0799 | Neutral |
| B116 | 87.98% | -0.067 | 0.284 | 0.0523 | Positive |
| D103 | 80.29% | -0.138 | 0.285 | 0.0221 | Neutral |
| B123 | 95.67% | 0.062 | 0.234 | 0.1865 | Neutral |
| D213 | 95.67% | -0.420 | 0.162 | -0.1265 | Balance |
| C4 | 93.75% | -0.115 | 0.346 | 0.0819 | Positive |
| SVA1 | 79.81% | -0.176 | 0.181 | 0.0869 | Neutral |
| SVA3 | 76.92% | -0.090 | 0.217 | 0.1007 | Neutral |
| SVA7 | 70.67% | -0.350 | 0.249 | -0.0666 | Neutral |
| SVA2 | 81.73% | 0.057 | 0.215 | 0.1084 | Neutral |
| SVA14 | 49.52% | -0.045 | 0.363 | 0.1137 | Positive |
| SVA15 | 94.23% | -0.380 | 0.160 | -0.1153 | Neutral |
| SVA4 | 72.12% | -0.215 | 0.232 | 0.0131 | Neutral |
| SVA12 | 41.83% | 0.195 | 0.444 | 0.2038 | Neutral |
| SVA16 | 60.58% | -0.140 | 0.391 | 0.0598 | Balance |
| SVA13 | 90.87% | -0.444 | 0.221 | -0.1311 | Neutral |
| SVA11 | 75.00% | -0.532 | 0.248 | -0.2239 | Balance |
| SVA10 | 70.67% | -0.263 | 0.206 | 0.0137 | Balance |
| SVA5 | 60.10% | -0.256 | 0.359 | -0.0728 | Balance |
| SVA18 | 77.40% | -0.047 | 0.276 | 0.1269 | Neutral |
| SVA8 | 59.62% | -0.076 | 0.236 | 0.1823 | Neutral |

Success, percentage of successfully-amplified individuals; *F_IS_*, overall *F_IS_* across the whole dataset; *F_ST_* overall *F_ST_* across the whole dataset; Oosterhout, null alleles frequency generated by Microchecker; Included/excluded, whether the loci included or excluded for following analysis; Selection, the selection situation of loci detected by LOSITAN.

Table S5. Basic population genetic information for the reduced microsatellite locus data set at three spatial scales for each of three species of deep-sea corals.

Table S5a. - Goniocorella dumosa

| Population | Locus | B106 | B1 | C107 | D5 | B123 | D213 | SVA13 | C4 | SVA15 | SVA10 | SVA2 | SVA18 | SVA8 | D103 | SVA1 | B116 | SVA11 | SVA4 | SVA7 | SVA20 | Mean | SE |
| --- | --- | --- | --- | --- | --- | --- | --- | --- | --- | --- | --- | --- | --- | --- | --- | --- | --- | --- | --- | --- | --- | --- | --- |
| Northern Province | N | 104 | 104 | 95 | 91 | 85 | 81 | 73 | 75 | 70 | 63 | 66 | 63 | 60 | 64 | 56 | 63 | 54 | 23 | 39 | 34 | 68.150 | 4.841 |
|  | Na | 7 | 7 | 15 | 16 | 12 | 14 | 12 | 15 | 25 | 9 | 13 | 22 | 20 | 10 | 11 | 13 | 7 | 16 | 15 | 17 | 13.800 | 1.085 |
|  | Ne | 4.258 | 2.125 | 4.926 | 8.658 | 3.898 | 5.295 | 4.786 | 4.987 | 6.644 | 5.005 | 3.546 | 6.005 | 4.343 | 6.015 | 3.942 | 5.682 | 2.633 | 11.500 | 8.895 | 6.404 | 5.477 | 0.494 |
|  | Ho | 0.808 | 0.904 | 0.547 | 0.769 | 0.706 | 0.790 | 0.877 | 0.613 | 0.629 | 0.730 | 0.652 | 0.667 | 0.750 | 0.703 | 0.339 | 0.651 | 1.000 | 0.913 | 0.641 | 0.824 | 0.726 | 0.033 |
|  | He | 0.765 | 0.529 | 0.797 | 0.884 | 0.743 | 0.811 | 0.791 | 0.799 | 0.849 | 0.800 | 0.718 | 0.833 | 0.770 | 0.834 | 0.746 | 0.824 | 0.620 | 0.913 | 0.888 | 0.844 | 0.788 | 0.020 |
|  | HWE | *** | *** | *** | ** | *** | *** | *** | *** | *** | *** | *** | *** | *** | *** | *** | *** | *** | ns | *** | ns |  |  |
| Southern Province | N | 4 | 4 | 4 | 4 | 3 | 3 | 3 | 3 | 2 | 2 | 2 | 2 | 1 | 2 | 1 | 1 | 3 | 0 | 0 | 1 | 2.250 | 0.289 |
|  | Na | 4 | 3 | 7 | 5 | 3 | 3 | 2 | 4 | 3 | 2 | 2 | 2 | 2 | 3 | 1 | 2 | 2 | 0 | 0 | 2 | 2.600 | 0.358 |
|  | Ne | 2.909 | 2.462 | 6.400 | 3.200 | 2.571 | 2.571 | 1.800 | 3.600 | 2.667 | 2.000 | 2.000 | 1.600 | 2.000 | 2.667 | 1.000 | 2.000 | 2.000 | 0.000 | 0.000 | 2.000 | 2.272 | 0.298 |
|  | Ho | 0.500 | 1.000 | 1.000 | 0.500 | 1.000 | 1.000 | 0.667 | 1.000 | 1.000 | 0.000 | 1.000 | 0.500 | 1.000 | 1.000 | 0.000 | 1.000 | 1.000 | 0.000 | 0.000 | 1.000 | 0.708 | 0.091 |
|  | He | 0.656 | 0.594 | 0.844 | 0.688 | 0.611 | 0.611 | 0.444 | 0.722 | 0.625 | 0.500 | 0.500 | 0.375 | 0.500 | 0.625 | 0.000 | 0.500 | 0.500 | 0.000 | 0.000 | 0.500 | 0.490 | 0.053 |
|  | HWE | ns | ns | ns | ns | ns | ns | ns | ns | ns | ns | ns | ns | ns | ns | Mono | ns | ns | Mono | Mono | ns |  |  |
| North  Region | N | 27 | 27 | 26 | 24 | 25 | 23 | 18 | 23 | 21 | 17 | 18 | 18 | 19 | 20 | 16 | 22 | 9 | 8 | 12 | 15 | 48.75 | 3.754 |
|  | Na | 7 | 5 | 10 | 13 | 10 | 10 | 10 | 11 | 17 | 6 | 10 | 12 | 14 | 8 | 10 | 10 | 3 | 9 | 9 | 11 | 11.500 | 0.838 |
|  | Ne | 4.942 | 2.236 | 4.012 | 7.024 | 4.209 | 5.264 | 6.353 | 4.301 | 7.056 | 5.352 | 5.945 | 6.231 | 4.813 | 5.634 | 5.505 | 5.068 | 2.219 | 7.529 | 5.538 | 6.818 | 5.074 | 0.486 |
|  | Ho | 0.778 | 0.963 | 0.500 | 0.708 | 1.000 | 0.739 | 0.833 | 0.652 | 0.714 | 0.824 | 0.889 | 0.778 | 0.842 | 0.800 | 0.625 | 0.636 | 1.000 | 1.000 | 0.500 | 0.933 | 0.698 | 0.037 |
|  | He | 0.798 | 0.553 | 0.751 | 0.858 | 0.762 | 0.810 | 0.843 | 0.767 | 0.858 | 0.813 | 0.832 | 0.840 | 0.792 | 0.823 | 0.818 | 0.803 | 0.549 | 0.867 | 0.819 | 0.853 | 0.768 | 0.022 |
|  | HWE | * | ** | *** | ns | * | ** | ns | ns | ns | ns | *** | ns | ns | ns | ns | ns | * | ns | ** | ns |  |  |
| Central  Region | N | 77 | 77 | 69 | 67 | 60 | 58 | 55 | 52 | 49 | 46 | 48 | 45 | 41 | 44 | 40 | 41 | 45 | 15 | 27 | 19 | 19.400 | 1.238 |
|  | Na | 6 | 6 | 13 | 16 | 11 | 11 | 10 | 12 | 17 | 8 | 8 | 19 | 15 | 10 | 9 | 12 | 6 | 15 | 14 | 12 | 9.750 | 0.695 |
|  | Ne | 3.836 | 2.084 | 4.972 | 8.96 | 3.752 | 4.745 | 4.133 | 5.026 | 6.141 | 4.575 | 2.753 | 5.556 | 3.979 | 5.779 | 2.747 | 5.827 | 2.682 | 10.465 | 8.526 | 4.945 | 5.303 | 0.321 |
|  | Ho | 0.818 | 0.883 | 0.565 | 0.791 | 0.583 | 0.81 | 0.891 | 0.596 | 0.592 | 0.696 | 0.563 | 0.622 | 0.707 | 0.659 | 0.225 | 0.659 | 1.000 | 0.867 | 0.704 | 0.737 | 0.786 | 0.034 |
|  | He | 0.739 | 0.52 | 0.799 | 0.888 | 0.733 | 0.789 | 0.758 | 0.801 | 0.837 | 0.781 | 0.637 | 0.820 | 0.749 | 0.827 | 0.636 | 0.828 | 0.627 | 0.904 | 0.883 | 0.798 | 0.790 | 0.020 |
|  | HWE | *** | *** | *** | *** | *** | *** | *** | *** | *** | *** | *** | *** | *** | *** | *** | *** | *** | ns | *** | ns |  |  |
| South  Region | N | 4 | 4 | 4 | 4 | 3 | 3 | 3 | 3 | 2 | 2 | 2 | 2 | 1 | 2 | 1 | 1 | 3 | 0 | 0 | 1 | 2.25 | 0.289 |
|  | Na | 4 | 3 | 7 | 5 | 3 | 3 | 2 | 4 | 3 | 2 | 2 | 2 | 2 | 3 | 1 | 2 | 2 | 0 | 0 | 2 | 2.6 | 0.358 |
|  | Ne | 2.909 | 2.462 | 6.400 | 3.200 | 2.571 | 2.571 | 1.800 | 3.600 | 2.667 | 2.000 | 2.000 | 1.600 | 2.000 | 2.667 | 1.000 | 2.000 | 2.000 | 0.000 | 0.000 | 2.000 | 2.272 | 0.298 |
|  | Ho | 0.500 | 1.000 | 1.000 | 0.500 | 1.000 | 1.000 | 0.667 | 1.000 | 1.000 | 0.000 | 1.000 | 0.500 | 1.000 | 1.000 | 0.000 | 1.000 | 1.000 | 0.000 | 0.000 | 1.000 | 0.708 | 0.091 |
|  | He | 0.656 | 0.594 | 0.844 | 0.688 | 0.611 | 0.611 | 0.444 | 0.722 | 0.625 | 0.500 | 0.500 | 0.375 | 0.500 | 0.625 | 0.000 | 0.500 | 0.500 | 0.000 | 0.000 | 0.500 | 0.490 | 0.053 |
|  | HWE | ns | ns | ns | ns | ns | ns | ns | ns | ns | ns | ns | ns | ns | ns | Mono | ns | ns | Mono | Mono | ns |  |  |
| Chatham Rise | N | 77 | 77 | 69 | 67 | 60 | 58 | 55 | 52 | 49 | 46 | 48 | 45 | 41 | 44 | 40 | 41 | 45 | 15 | 27 | 19 | 48.750 | 3.754 |
|  | Na | 6 | 6 | 13 | 16 | 11 | 11 | 10 | 12 | 17 | 8 | 8 | 19 | 15 | 10 | 9 | 12 | 6 | 15 | 14 | 12 | 11.500 | 0.838 |
|  | Ne | 3.836 | 2.084 | 4.972 | 8.96 | 3.752 | 4.745 | 4.133 | 5.026 | 6.141 | 4.575 | 2.753 | 5.556 | 3.979 | 5.779 | 2.747 | 5.827 | 2.682 | 10.465 | 8.526 | 4.945 | 5.074 | 0.486 |
|  | Ho | 0.818 | 0.883 | 0.565 | 0.791 | 0.583 | 0.81 | 0.891 | 0.596 | 0.592 | 0.696 | 0.563 | 0.622 | 0.707 | 0.659 | 0.225 | 0.659 | 1.000 | 0.867 | 0.704 | 0.737 | 0.698 | 0.037 |
|  | He | 0.739 | 0.52 | 0.799 | 0.888 | 0.733 | 0.789 | 0.758 | 0.801 | 0.837 | 0.781 | 0.637 | 0.820 | 0.749 | 0.827 | 0.636 | 0.828 | 0.627 | 0.904 | 0.883 | 0.798 | 0.768 | 0.022 |
|  | HWE | *** | *** | *** | *** | *** | *** | *** | *** | *** | *** | *** | *** | *** | *** | *** | *** | *** | ns | *** | ns |  |  |
| Kermadec Ridge | N | 12 | 12 | 12 | 11 | 12 | 12 | 8 | 12 | 9 | 8 | 10 | 9 | 10 | 11 | 9 | 12 | 3 | 3 | 5 | 7 | 9.350 | 0.658 |
|  | Na | 6 | 2 | 9 | 9 | 9 | 6 | 5 | 8 | 11 | 6 | 10 | 10 | 9 | 7 | 6 | 8 | 2 | 5 | 4 | 7 | 6.905 | 0.569 |
|  | Ne | 4.114 | 1.986 | 3.840 | 4.939 | 4.571 | 4.114 | 3.879 | 4.114 | 8.100 | 4.923 | 7.143 | 5.786 | 3.509 | 4.654 | 3.682 | 4.114 | 2.000 | 4.500 | 2.941 | 5.765 | 4.434 | 0.331 |
|  | Ho | 0.750 | 0.917 | 0.500 | 0.455 | 1.000 | 0.750 | 0.750 | 0.750 | 0.889 | 0.750 | 0.800 | 0.889 | 0.700 | 0.818 | 0.556 | 0.667 | 1.000 | 1.000 | 0.200 | 1.000 | 0.757 | 0.047 |
|  | He | 0.757 | 0.497 | 0.740 | 0.798 | 0.781 | 0.757 | 0.742 | 0.757 | 0.877 | 0.797 | 0.860 | 0.827 | 0.715 | 0.785 | 0.728 | 0.757 | 0.500 | 0.778 | 0.660 | 0.827 | 0.747 | 0.022 |
|  | HWE | ns | ** | * | ** | ns | ns | ns | ns | ns | ns | ns | ns | ns | ns | ns | ns | ns | ns | * | ns |  |  |
| Challenger Plateau | N | 7 | 7 | 7 | 7 | 6 | 4 | 5 | 5 | 5 | 5 | 3 | 4 | 4 | 4 | 5 | 5 | 3 | 3 | 1 | 4 | 4.700 | 0.356 |
|  | Na | 5 | 4 | 4 | 7 | 5 | 4 | 7 | 7 | 6 | 6 | 4 | 5 | 6 | 6 | 7 | 4 | 3 | 4 | 1 | 5 | 5.000 | 0.348 |
|  | Ne | 4.455 | 2.579 | 2.513 | 5.444 | 3.600 | 3.200 | 6.250 | 5.556 | 4.167 | 5.556 | 3.600 | 4.000 | 4.571 | 4.571 | 6.250 | 3.846 | 2.571 | 3.600 | 1.000 | 3.200 | 4.026 | 0.303 |
|  | Ho | 0.714 | 1.000 | 0.571 | 1.000 | 1.000 | 0.500 | 1.000 | 0.600 | 0.400 | 1.000 | 1.000 | 0.750 | 1.000 | 1.000 | 0.800 | 0.600 | 1.000 | 1.000 | 0.000 | 1.000 | 0.797 | 0.062 |
|  | He | 0.776 | 0.612 | 0.602 | 0.816 | 0.722 | 0.688 | 0.840 | 0.820 | 0.760 | 0.820 | 0.722 | 0.750 | 0.781 | 0.781 | 0.840 | 0.740 | 0.611 | 0.722 | 0.000 | 0.688 | 0.705 | 0.041 |
|  | HWE | ns | ns | ns | ns | ns | ns | ns | ns | * | ns | ns | ns | ns | ns | ns | ns | ns | ns | Mono | ns |  |  |
| NE continental slope | N | 4 | 4 | 3 | 3 | 4 | 4 | 3 | 4 | 4 | 3 | 3 | 4 | 3 | 3 | 1 | 3 | 3 | 0 | 4 | 3 | 3.150 | 0.233 |
|  | Na | 5.000 | 3.000 | 4.000 | 4.000 | 4.000 | 6.000 | 3.000 | 3.000 | 7.000 | 3.000 | 3.000 | 2.000 | 5.000 | 3.000 | 1.000 | 4.000 | 2.000 | 0.000 | 5.000 | 4.000 | 3.550 | 0.366 |
|  | Ne | 4.000 | 2.462 | 3.600 | 3.000 | 2.909 | 5.333 | 2.571 | 1.684 | 6.400 | 2.571 | 2.571 | 2.000 | 4.500 | 3.000 | 1.000 | 3.600 | 2.000 | 0.000 | 4.571 | 3.000 | 3.039 | 0.328 |
|  | Ho | 1.000 | 1.000 | 0.333 | 0.667 | 1.000 | 1.000 | 1.000 | 0.500 | 1.000 | 0.667 | 1.000 | 0.500 | 1.000 | 0.667 | 0.000 | 0.333 | 1.000 | 0.000 | 1.000 | 0.667 | 0.717 | 0.077 |
|  | He | 0.750 | 0.594 | 0.722 | 0.667 | 0.656 | 0.813 | 0.611 | 0.406 | 0.844 | 0.611 | 0.611 | 0.500 | 0.778 | 0.667 | 0.000 | 0.722 | 0.500 | 0.000 | 0.781 | 0.667 | 0.595 | 0.052 |
|  | HWE | ns | ns | ns | ns | ns | ns | ns | ns | * | ns | ns | ns | ns | ns | Mono | ns | ns | Mono | ns | ns |  |  |

Table S5b. - Madrepora oculata

| Population | Locus | c016 | g028 | c7 | g006 | g004 | g25 | c013 | g016 | g022 | a5 | g010 | Mean | SE |
| --- | --- | --- | --- | --- | --- | --- | --- | --- | --- | --- | --- | --- | --- | --- |
| Northern Province | N | 46 | 37 | 20 | 42 | 45 | 22 | 54 | 24 | 23 | 41 | 45 | 36.273 | 3.583 |
|  | Na | 14 | 18 | 13 | 11 | 16 | 5 | 11 | 14 | 15 | 18 | 13 | 13.455 | 1.107 |
|  | Ne | 9.51 | 8.322 | 5.442 | 5.219 | 10.385 | 4.19 | 2.602 | 5.62 | 5.75 | 4.344 | 6.853 | 0.814 | 0.024 |
|  | Ho | 0.891 | 0.757 | 0.7 | 0.738 | 0.622 | 0.273 | 0.463 | 0.708 | 0.478 | 0.488 | 0.689 | 0.619 | 0.053 |
|  | He | 0.895 | 0.88 | 0.816 | 0.808 | 0.904 | 0.761 | 0.616 | 0.822 | 0.826 | 0.77 | 0.854 | 0.814 | 0.024 |
|  | HWE | ** | *** | *** | *** | *** | *** | *** | ** | *** | *** | *** |  |  |
| Southern Province | N | 15 | 7 | 6 | 10 | 15 | 1 | 14 | 4 | 5 | 11 | 12 | 9.091 | 1.443 |
|  | Na | 11 | 6 | 5 | 4 | 13 | 1 | 4 | 4 | 5 | 9 | 9 | 6.455 | 1.082 |
|  | Ne | 6.818 | 4.261 | 3.789 | 3.39 | 8.036 | 1 | 2.78 | 2.909 | 3.125 | 5.902 | 4.364 | 0.683 | 0.072 |
|  | Ho | 0.867 | 0.857 | 0.833 | 0.7 | 0.733 | 0 | 0.643 | 0.75 | 0.4 | 0.455 | 0.667 | 0.628 | 0.078 |
|  | He | 0.853 | 0.765 | 0.736 | 0.705 | 0.876 | 0 | 0.64 | 0.656 | 0.68 | 0.831 | 0.771 | 0.683 | 0.072 |
|  | HWE | * | ns | ns | ns | * | Mono | * | ns | ns | *** | ** |  |  |
| North Region | N | 18 | 15 | 6 | 13 | 15 | 9 | 16 | 8 | 9 | 15 | 14 | 12.545 | 1.171 |
|  | Na | 14 | 14 | 8 | 8 | 11 | 5 | 7 | 8 | 6 | 13 | 11 | 9.545 | 0.966 |
|  | Ne | 9.127 | 9.184 | 6.545 | 5.2 | 7.5 | 4.378 | 2.197 | 4.923 | 3.375 | 6.522 | 6.323 | 5.934 | 0.664 |
|  | Ho | 0.944 | 0.8 | 0.667 | 0.615 | 0.733 | 0.111 | 0.438 | 0.625 | 0.444 | 0.533 | 0.786 | 0.609 | 0.068 |
|  | He | 0.89 | 0.891 | 0.847 | 0.808 | 0.867 | 0.772 | 0.545 | 0.797 | 0.704 | 0.847 | 0.842 | 0.801 | 0.03 |
|  | HWE | ns | ns | ns | ** | ns | ** | ns | ns | * | *** | ns |  |  |
| Central Region | N | 30 | 23 | 15 | 30 | 32 | 13 | 39 | 16 | 15 | 28 | 33 | 24.909 | 2.692 |
|  | Na | 12 | 14 | 9 | 8 | 16 | 5 | 10 | 12 | 11 | 14 | 11 | 11.091 | 0.929 |
|  | Ne | 8.145 | 6.491 | 4.018 | 4.945 | 9.752 | 3.25 | 2.873 | 5.447 | 6.338 | 3.401 | 6.6 | 5.569 | 0.652 |
|  | Ho | 0.867 | 0.739 | 0.733 | 0.8 | 0.563 | 0.385 | 0.462 | 0.75 | 0.467 | 0.464 | 0.636 | 0.624 | 0.049 |
|  | He | 0.877 | 0.846 | 0.751 | 0.798 | 0.897 | 0.692 | 0.652 | 0.816 | 0.842 | 0.706 | 0.848 | 0.793 | 0.025 |
|  | HWE | ** | ns | * | *** | *** | *** | *** | ns | ** | *** | *** |  |  |
| South Region | N | 13 | 6 | 5 | 9 | 13 | 1 | 13 | 4 | 4 | 9 | 10 | 7.909 | 1.261 |
|  | Na | 8 | 6 | 5 | 4 | 12 | 1 | 3 | 4 | 5 | 8 | 9 | 5.909 | 0.939 |
|  | Ne | 6.818 | 4.261 | 3.789 | 3.39 | 8.036 | 1 | 2.78 | 2.909 | 3.125 | 5.902 | 4.364 | 0.683 | 0.072 |
|  | Ho | 0.846 | 0.833 | 0.8 | 0.667 | 0.769 | 0 | 0.692 | 0.75 | 0.5 | 0.444 | 0.7 | 0.637 | 0.074 |
|  | He | 0.825 | 0.778 | 0.74 | 0.698 | 0.876 | 0 | 0.589 | 0.656 | 0.75 | 0.84 | 0.815 | 0.688 | 0.073 |
|  | HWE | ns | ns | ns | ns | * | Mono | ns | ns | ns | *** | * |  |  |
| Chatham Rise | N | 30 | 23 | 15 | 30 | 32 | 13 | 39 | 16 | 15 | 28 | 33 | 4.727 | 0.648 |
|  | Na | 12 | 14 | 9 | 8 | 16 | 5 | 10 | 12 | 11 | 14 | 11 | 5.364 | 0.812 |
|  | Ne | 8.145 | 6.491 | 4.018 | 4.945 | 9.752 | 3.25 | 2.873 | 5.447 | 6.338 | 3.401 | 6.6 | 5.569 | 0.652 |
|  | Ho | 0.867 | 0.739 | 0.733 | 0.8 | 0.563 | 0.385 | 0.462 | 0.75 | 0.467 | 0.464 | 0.636 | 0.623 | 0.106 |
|  | He | 0.877 | 0.846 | 0.751 | 0.798 | 0.897 | 0.692 | 0.652 | 0.816 | 0.842 | 0.706 | 0.848 | 0.689 | 0.053 |
|  | HWE | ** | ns | * | *** | *** | *** | *** | ns | ** | *** | *** |  |  |
| Campbell Plateau | N | 14 | 7 | 6 | 9 | 15 | 1 | 14 | 4 | 4 | 10 | 11 | 8.636 | 1.403 |
|  | Na | 11 | 6 | 5 | 4 | 13 | 1 | 4 | 4 | 5 | 8 | 9 | 6.364 | 1.064 |
|  | Ne | 7.259 | 4.261 | 3.789 | 3.176 | 8.036 | 1.000 | 2.780 | 2.909 | 4.000 | 5.128 | 3.967 | 4.210 | 0.604 |
|  | Ho | 0.857 | 0.857 | 0.833 | 0.667 | 0.733 | 0.000 | 0.643 | 0.750 | 0.500 | 0.500 | 0.636 | 0.634 | 0.074 |
|  | He | 0.862 | 0.765 | 0.736 | 0.685 | 0.876 | 0.000 | 0.640 | 0.656 | 0.750 | 0.805 | 0.748 | 0.684 | 0.072 |
|  | HWE | * | ns | ns | ns | * | Mono | * | ns | ns | ** | * |  |  |
| Challenger Plateau | N | 8 | 6 | 3 | 9 | 6 | 6 | 6 | 4 | 5 | 9 | 8 | 6.364 | 0.592 |
|  | Na | 9 | 7 | 6 | 6 | 6 | 3 | 6 | 4 | 2 | 11 | 8 | 6.182 | 0.784 |
|  | Ne | 5.333 | 6.000 | 6.000 | 4.765 | 4.500 | 2.571 | 2.667 | 2.909 | 1.471 | 10.125 | 4.129 | 4.588 | 0.712 |
|  | Ho | 1.000 | 0.833 | 1.000 | 0.444 | 0.500 | 0.000 | 0.500 | 1.000 | 0.400 | 0.556 | 0.750 | 0.635 | 0.095 |
|  | He | 0.813 | 0.833 | 0.833 | 0.790 | 0.778 | 0.611 | 0.625 | 0.656 | 0.320 | 0.901 | 0.758 | 0.720 | 0.049 |
|  | HWE | ns | ns | ns | * | ns | ** | ns | ns | ns | * | ns |  |  |
| Kermadec Ridge | N | 8 | 7 | 2 | 3 | 6 | 3 | 8 | 4 | 3 | 4 | 4 | 4.727 | 0.648 |
|  | Na | 10 | 10 | 2 | 5 | 6 | 3 | 3 | 5 | 4 | 4 | 7 | 5.364 | 0.812 |
|  | Ne | 8.533 | 8.167 | 2.000 | 4.500 | 4.000 | 2.571 | 1.471 | 4.571 | 3.000 | 2.286 | 6.400 | 4.318 | 0.736 |
|  | Ho | 0.875 | 0.857 | 0.000 | 1.000 | 1.000 | 0.333 | 0.375 | 0.250 | 0.667 | 0.500 | 1.000 | 0.623 | 0.106 |
|  | He | 0.883 | 0.878 | 0.500 | 0.778 | 0.750 | 0.611 | 0.320 | 0.781 | 0.667 | 0.563 | 0.844 | 0.689 | 0.053 |
|  | HWE | ns | ns | ns | ns | ns | ns | ns | ns | ns | ns | ns |  |  |

Table S5c. - Solenosmilia variabilis

| Population | Locus | B106 | C107 | D5 | B1 | B116 | D103 | B123 | D213 | C4 | SVA1 | SVA3 | SVA7 | SVA2 | SVA14 | SVA15 | SVA4 | SVA12 | SVA16 | SVA13 | SVA11 | SVA10 | SVA5 | SVA18 | SVA8 | Mean | SE |
| --- | --- | --- | --- | --- | --- | --- | --- | --- | --- | --- | --- | --- | --- | --- | --- | --- | --- | --- | --- | --- | --- | --- | --- | --- | --- | --- | --- |
| Northern Province | N | 163 | 159 | 160 | 162 | 148 | 135 | 161 | 160 | 159 | 132 | 132 | 123 | 133 | 88 | 159 | 117 | 70 | 102 | 153 | 126 | 119 | 102 | 133 | 101 | 133.208 | 5.414 |
|  | Na | 9 | 17 | 19 | 19 | 14 | 7 | 13 | 22 | 11 | 19 | 22 | 18 | 12 | 22 | 22 | 26 | 20 | 13 | 15 | 22 | 23 | 12 | 31 | 26 | 18.083 | 1.21 |
|  | Ne | 2.632 | 4.28 | 5.411 | 4.661 | 3.932 | 2.331 | 1.979 | 5.156 | 1.398 | 3.242 | 6.333 | 5.887 | 6.676 | 14.821 | 3.956 | 8.197 | 5.847 | 2.459 | 2.689 | 3.967 | 9.824 | 7.368 | 7.125 | 8.064 | 5.343 | 0.612 |
|  | Ho | 0.957 | 0.736 | 0.788 | 0.901 | 0.682 | 0.548 | 0.304 | 0.988 | 0.245 | 0.598 | 0.659 | 0.943 | 0.677 | 0.727 | 0.887 | 0.846 | 0.471 | 0.539 | 0.732 | 0.984 | 0.882 | 0.98 | 0.639 | 0.545 | 0.719 | 0.043 |
|  | He | 0.62 | 0.766 | 0.815 | 0.785 | 0.746 | 0.571 | 0.495 | 0.806 | 0.285 | 0.692 | 0.842 | 0.830 | 0.850 | 0.933 | 0.747 | 0.878 | 0.829 | 0.593 | 0.628 | 0.748 | 0.898 | 0.864 | 0.860 | 0.876 | 0.748 | 0.031 |
|  | HWE | *** | *** | *** | *** | *** | *** | *** | *** | *** | *** | *** | *** | *** | *** | *** | *** | *** | *** | *** | *** | *** | *** | *** | *** |  |  |
| Southern Province | N | 38 | 37 | 38 | 39 | 35 | 32 | 38 | 39 | 36 | 34 | 28 | 24 | 37 | 15 | 37 | 33 | 17 | 24 | 36 | 30 | 28 | 23 | 28 | 23 | 31.208 | 1.455 |
|  | Na | 5 | 12 | 13 | 11 | 7 | 5 | 9 | 12 | 6 | 12 | 11 | 10 | 9 | 13 | 13 | 18 | 12 | 10 | 7 | 8 | 14 | 10 | 14 | 17 | 10.750 | 0.695 |
|  | Ne | 2.283 | 5.099 | 6.639 | 3.441 | 3.895 | 3.205 | 1.807 | 4.5 | 1.624 | 2.953 | 6.817 | 7.432 | 4.265 | 9.375 | 5.024 | 10.573 | 7.049 | 3.282 | 2.374 | 2.932 | 8.909 | 7.149 | 6.730 | 10.373 | 5.322 | 0.556 |
|  | Ho | 0.895 | 0.946 | 0.737 | 0.744 | 0.514 | 0.625 | 0.289 | 0.949 | 0.25 | 0.588 | 0.75 | 0.917 | 0.595 | 0.667 | 0.892 | 0.909 | 0.588 | 0.583 | 0.778 | 1.000 | 0.857 | 1.000 | 0.643 | 0.696 | 0.725 | 0.042 |
|  | He | 0.562 | 0.804 | 0.849 | 0.709 | 0.743 | 0.688 | 0.447 | 0.778 | 0.384 | 0.661 | 0.853 | 0.865 | 0.766 | 0.893 | 0.801 | 0.905 | 0.858 | 0.695 | 0.579 | 0.659 | 0.888 | 0.860 | 0.851 | 0.904 | 0.750 | 0.029 |
|  | HWE | *** | *** | * | *** | *** | ** | *** | *** | *** | *** | ns | ns | ns | ns | *** | *** | ** | *** | *** | *** | * | *** | *** | ns |  |  |
| North  Region | N | 80 | 79 | 79 | 79 | 70 | 68 | 80 | 77 | 80 | 71 | 77 | 74 | 72 | 53 | 80 | 60 | 38 | 58 | 76 | 64 | 69 | 56 | 72 | 53 | 69.375 | 2.282 |
|  | Na | 8 | 13 | 18 | 17 | 13 | 7 | 12 | 21 | 8 | 18 | 16 | 16 | 10 | 21 | 14 | 16 | 14 | 11 | 15 | 18 | 21 | 11 | 23 | 23 | 15.167 | 0.959 |
|  | Ne | 3.197 | 4.411 | 5.33 | 7.048 | 4.048 | 2.169 | 2.162 | 6.566 | 1.298 | 4.211 | 5.01 | 5.77 | 7.175 | 13.872 | 3.514 | 7.385 | 7.166 | 2.475 | 3.447 | 5.543 | 10.206 | 7.024 | 7.145 | 8.946 | 5.630 | 0.585 |
|  | Ho | 0.925 | 0.709 | 0.785 | 0.962 | 0.729 | 0.5 | 0.313 | 0.987 | 0.213 | 0.648 | 0.623 | 0.959 | 0.708 | 0.868 | 0.900 | 0.783 | 0.553 | 0.534 | 0.737 | 0.984 | 0.899 | 0.964 | 0.681 | 0.604 | 0.732 | 0.043 |
|  | He | 0.687 | 0.773 | 0.812 | 0.858 | 0.753 | 0.539 | 0.538 | 0.848 | 0.23 | 0.763 | 0.800 | 0.827 | 0.861 | 0.928 | 0.715 | 0.865 | 0.86 | 0.596 | 0.710 | 0.820 | 0.902 | 0.858 | 0.860 | 0.888 | 0.762 | 0.032 |
|  | HWE | *** | *** | *** | *** | *** | *** | *** | *** | *** | *** | *** | *** | *** | *** | *** | *** | *** | *** | *** | *** | *** | *** | *** | *** |  |  |
| Central  Region | N | 83 | 80 | 81 | 83 | 78 | 67 | 81 | 83 | 79 | 61 | 55 | 49 | 61 | 35 | 79 | 57 | 32 | 44 | 77 | 62 | 50 | 46 | 61 | 48 | 63.833 | 3.343 |
|  | Na | 7 | 14 | 14 | 13 | 9 | 6 | 10 | 9 | 7 | 12 | 18 | 12 | 12 | 18 | 18 | 22 | 13 | 11 | 8 | 13 | 17 | 11 | 23 | 21 | 13.25 | 0.984 |
|  | Ne | 2.176 | 4.057 | 5.166 | 3.191 | 3.672 | 2.483 | 1.808 | 4.135 | 1.505 | 2.442 | 8.013 | 5.965 | 5.742 | 11.557 | 4.292 | 8.483 | 4.163 | 2.344 | 2.103 | 2.815 | 8.803 | 7.425 | 6.615 | 6.847 | 4.825 | 0.543 |
|  | Ho | 0.988 | 0.763 | 0.79 | 0.843 | 0.641 | 0.597 | 0.296 | 0.988 | 0.278 | 0.541 | 0.709 | 0.918 | 0.639 | 0.514 | 0.873 | 0.912 | 0.375 | 0.545 | 0.727 | 0.984 | 0.860 | 1.000 | 0.590 | 0.479 | 0.702 | 0.045 |
|  | He | 0.54 | 0.754 | 0.806 | 0.687 | 0.728 | 0.597 | 0.447 | 0.758 | 0.335 | 0.59 | 0.875 | 0.832 | 0.826 | 0.913 | 0.767 | 0.882 | 0.76 | 0.573 | 0.524 | 0.645 | 0.886 | 0.865 | 0.849 | 0.854 | 0.721 | 0.032 |
|  | HWE | *** | *** | *** | *** | *** | *** | *** | *** | *** | ** | *** | *** | *** | *** | *** | *** | *** | *** | *** | *** | *** | *** | *** | *** |  |  |
| South  Region | N | 38 | 37 | 38 | 39 | 35 | 32 | 38 | 39 | 36 | 34 | 28 | 24 | 37 | 15 | 37 | 33 | 17 | 24 | 36 | 30 | 28 | 23 | 28 | 23 | 31.208 | 1.455 |
|  | Na | 5 | 12 | 13 | 11 | 7 | 5 | 9 | 12 | 6 | 12 | 11 | 10 | 9 | 13 | 13 | 18 | 12 | 10 | 7 | 8 | 14 | 10 | 14 | 17 | 10.750 | 0.695 |
|  | Ne | 2.283 | 5.099 | 6.639 | 3.441 | 3.895 | 3.205 | 1.807 | 4.5 | 1.624 | 2.953 | 6.817 | 7.432 | 4.265 | 9.375 | 5.024 | 10.573 | 7.049 | 3.282 | 2.374 | 2.932 | 8.909 | 7.149 | 6.73 | 10.373 | 5.322 | 0.556 |
|  | Ho | 0.895 | 0.946 | 0.737 | 0.744 | 0.514 | 0.625 | 0.289 | 0.949 | 0.25 | 0.588 | 0.75 | 0.917 | 0.595 | 0.667 | 0.892 | 0.909 | 0.588 | 0.583 | 0.778 | 1.000 | 0.857 | 1.000 | 0.643 | 0.696 | 0.725 | 0.042 |
|  | He | 0.562 | 0.804 | 0.849 | 0.709 | 0.743 | 0.688 | 0.447 | 0.778 | 0.384 | 0.661 | 0.853 | 0.865 | 0.766 | 0.893 | 0.801 | 0.905 | 0.858 | 0.695 | 0.579 | 0.659 | 0.888 | 0.86 | 0.851 | 0.904 | 0.750 | 0.029 |
|  | HWE | *** | *** | * | *** | *** | ** | *** | *** | *** | *** | ns | ns | ns | ns | *** | *** | ** | *** | *** | *** | * | *** | *** | ns |  |  |
| Chatham Rise | N | 83 | 80 | 81 | 83 | 78 | 67 | 81 | 83 | 79 | 61 | 55 | 49 | 61 | 35 | 79 | 57 | 32 | 44 | 77 | 62 | 50 | 46 | 61 | 48 | 63.833 | 3.343 |
|  | Na | 7 | 14 | 14 | 13 | 9 | 6 | 10 | 9 | 7 | 12 | 18 | 12 | 12 | 18 | 18 | 22 | 13 | 11 | 8 | 13 | 17 | 11 | 23 | 21 | 13.250 | 0.984 |
|  | Ne | 2.176 | 4.057 | 5.166 | 3.191 | 3.672 | 2.483 | 1.808 | 4.135 | 1.505 | 2.442 | 8.013 | 5.965 | 5.742 | 11.557 | 4.292 | 8.483 | 4.163 | 2.344 | 2.103 | 2.815 | 8.803 | 7.425 | 6.615 | 6.847 | 4.825 | 0.543 |
|  | Ho | 0.988 | 0.763 | 0.79 | 0.843 | 0.641 | 0.597 | 0.296 | 0.988 | 0.278 | 0.541 | 0.709 | 0.918 | 0.639 | 0.514 | 0.873 | 0.912 | 0.375 | 0.545 | 0.727 | 0.984 | 0.860 | 1.000 | 0.590 | 0.479 | 0.702 | 0.045 |
|  | He | 0.54 | 0.754 | 0.806 | 0.687 | 0.728 | 0.597 | 0.447 | 0.758 | 0.335 | 0.59 | 0.875 | 0.832 | 0.826 | 0.913 | 0.767 | 0.882 | 0.76 | 0.573 | 0.524 | 0.645 | 0.886 | 0.865 | 0.849 | 0.854 | 0.721 | 0.032 |
|  | HWE | *** | *** | *** | *** | *** | *** | *** | *** | *** | ** | *** | *** | *** | *** | *** | *** | *** | *** | *** | *** | *** | *** | *** | *** |  |  |
| Kermadec Ridge | N | 40 | 39 | 39 | 39 | 35 | 33 | 39 | 40 | 38 | 35 | 36 | 35 | 34 | 28 | 39 | 31 | 21 | 31 | 37 | 28 | 33 | 30 | 36 | 28 | 34.333 | 0.992 |
|  | Na | 7 | 9 | 15 | 15 | 8 | 7 | 10 | 17 | 7 | 10 | 12 | 12 | 9 | 18 | 12 | 14 | 7 | 8 | 12 | 9 | 16 | 11 | 18 | 17 | 11.667 | 0.768 |
|  | Ne | 2.576 | 4.702 | 4.061 | 7.141 | 2.984 | 2.679 | 2.227 | 4.769 | 1.282 | 1.989 | 4.662 | 6.447 | 6.165 | 13.635 | 3.723 | 7.364 | 4.594 | 2.379 | 3.271 | 2.872 | 8.250 | 7.004 | 6.529 | 8.385 | 4.987 | 0.571 |
|  | Ho | 0.925 | 0.795 | 0.744 | 0.923 | 0.629 | 0.515 | 0.282 | 0.975 | 0.211 | 0.486 | 0.639 | 1.000 | 0.735 | 0.857 | 0.949 | 0.839 | 0.476 | 0.548 | 0.811 | 1.000 | 1.000 | 0.933 | 0.667 | 0.571 | 0.730 | 0.047 |
|  | He | 0.612 | 0.787 | 0.754 | 0.86 | 0.665 | 0.627 | 0.551 | 0.79 | 0.22 | 0.497 | 0.785 | 0.845 | 0.838 | 0.927 | 0.731 | 0.864 | 0.782 | 0.58 | 0.694 | 0.652 | 0.879 | 0.857 | 0.847 | 0.881 | 0.730 | 0.033 |
|  | HWE | * | *** | *** | *** | ** | *** | *** | *** | *** | *** | *** | *** | ns | ns | *** | ** | *** | *** | *** | *** | *** | ns | *** | ** |  |  |
| Louisville Seamount Chain | N | 35 | 35 | 35 | 35 | 30 | 30 | 36 | 32 | 37 | 31 | 36 | 34 | 34 | 23 | 36 | 25 | 13 | 23 | 34 | 32 | 32 | 23 | 31 | 22 | 30.583 | 1.231 |
|  | Na | 6 | 9 | 13 | 12 | 10 | 5 | 8 | 15 | 4 | 16 | 13 | 11 | 10 | 15 | 11 | 9 | 10 | 8 | 11 | 15 | 15 | 9 | 15 | 15 | 11.042 | 0.703 |
|  | Ne | 4.077 | 3.137 | 5.889 | 5.53 | 4.455 | 1.654 | 1.799 | 9.143 | 1.284 | 9.856 | 3.216 | 4.817 | 5.766 | 9.281 | 3.256 | 6.250 | 5.729 | 2.625 | 3.676 | 8.569 | 5.721 | 5.688 | 6.744 | 6.769 | 5.205 | 0.495 |
|  | Ho | 0.943 | 0.571 | 0.8 | 1.000 | 0.833 | 0.433 | 0.278 | 1.000 | 0.189 | 0.871 | 0.583 | 0.912 | 0.735 | 0.870 | 0.833 | 0.720 | 0.692 | 0.522 | 0.676 | 0.969 | 0.781 | 1.000 | 0.710 | 0.591 | 0.730 | 0.045 |
|  | He | 0.755 | 0.681 | 0.83 | 0.819 | 0.776 | 0.396 | 0.444 | 0.891 | 0.221 | 0.899 | 0.689 | 0.792 | 0.827 | 0.892 | 0.693 | 0.840 | 0.825 | 0.619 | 0.728 | 0.883 | 0.825 | 0.824 | 0.852 | 0.852 | 0.744 | 0.035 |
|  | HWE | *** | ns | *** | *** | *** | ns | *** | *** | *** | *** | *** | *** | *** | ns | ns | * | *** | *** | ns | ** | *** | ns | ns | *** |  |  |
| Macquarie Ridge | N | 15 | 13 | 15 | 15 | 13 | 12 | 14 | 15 | 13 | 11 | 7 | 6 | 15 | 2 | 14 | 12 | 6 | 7 | 14 | 10 | 8 | 6 | 8 | 4 | 10.625 | 0.823 |
|  | Na | 2 | 8 | 10 | 6 | 5 | 5 | 5 | 7 | 5 | 6 | 7 | 6 | 6 | 3 | 9 | 10 | 8 | 6 | 3 | 3 | 7 | 8 | 7 | 3 | 6.042 | 0.452 |
|  | Ne | 2.000 | 4.390 | 7.143 | 2.813 | 3.976 | 3.310 | 1.867 | 4.245 | 1.965 | 2.521 | 5.444 | 4.800 | 3.516 | 2.667 | 5.851 | 8.727 | 6.545 | 4.667 | 2.074 | 2.198 | 5.333 | 6.545 | 6.095 | 2.667 | 4.223 | 0.391 |
|  | Ho | 1.000 | 0.923 | 0.867 | 1.000 | 0.462 | 0.583 | 0.286 | 1.000 | 0.385 | 0.636 | 0.714 | 1.000 | 0.467 | 0.500 | 0.786 | 0.833 | 0.667 | 0.571 | 0.857 | 1.000 | 0.750 | 1.000 | 0.750 | 0.000 | 0.71 | 0.054 |
|  | He | 0.500 | 0.772 | 0.860 | 0.644 | 0.749 | 0.698 | 0.464 | 0.764 | 0.491 | 0.603 | 0.816 | 0.792 | 0.716 | 0.625 | 0.829 | 0.885 | 0.847 | 0.786 | 0.518 | 0.545 | 0.813 | 0.847 | 0.836 | 0.625 | 0.709 | 0.027 |
|  | HWE | *** | * | ns | *** | ** | * | ns | *** | ns | ns | ns | ns | * | ns | *** | ** | ns | ns | * | * | ns | ns | ns | * |  |  |
| Bounty Trough | N | 5 | 5 | 5 | 5 | 5 | 5 | 5 | 5 | 5 | 5 | 5 | 4 | 5 | 4 | 5 | 5 | 3 | 5 | 5 | 4 | 4 | 5 | 5 | 4 | 4.708 | 0.112 |
|  | Na | 3 | 5 | 5 | 5 | 4 | 4 | 2 | 5 | 3 | 5 | 6 | 5 | 4 | 5 | 2 | 6 | 3 | 7 | 3 | 3 | 5 | 6 | 6 | 5 | 4.458 | 0.276 |
|  | Ne | 2.381 | 3.571 | 3.571 | 3.846 | 3.333 | 2.381 | 1.220 | 3.571 | 1.515 | 2.500 | 4.545 | 4.000 | 3.846 | 3.200 | 2.000 | 5.556 | 2.571 | 4.545 | 1.852 | 2.462 | 4.000 | 5.000 | 4.167 | 4.571 | 2.381 | 0.233 |
|  | Ho | 1.000 | 1.000 | 0.600 | 0.600 | 0.600 | 0.400 | 0.200 | 1.000 | 0.400 | 0.600 | 0.800 | 1.000 | 0.400 | 0.750 | 1.000 | 1.000 | 0.333 | 0.800 | 0.600 | 1.000 | 1.000 | 1.000 | 0.600 | 0.500 | 1.000 | 0.054 |
|  | He | 0.580 | 0.720 | 0.720 | 0.740 | 0.700 | 0.580 | 0.180 | 0.720 | 0.340 | 0.600 | 0.780 | 0.750 | 0.740 | 0.688 | 0.500 | 0.820 | 0.611 | 0.780 | 0.460 | 0.594 | 0.750 | 0.800 | 0.760 | 0.781 | 0.580 | 0.032 |
|  | HWE | ns | ns | ns | ns | ns | ns | ns | ns | ns | ns | ns | ns | ns | ns | ns | ns | ns | ns | ns | ns | ns | ns | ns | ns |  |  |
| Bounty Plateau | N | 4 | 4 | 4 | 4 | 4 | 4 | 4 | 4 | 4 | 4 | 4 | 4 | 4 | 3 | 4 | 4 | 3 | 4 | 4 | 4 | 4 | 4 | 4 | 4 | 3.917 | 0.058 |
|  | Na | 3.000 | 3.000 | 4.000 | 4.000 | 2.000 | 4.000 | 2.000 | 2.000 | 1.000 | 3.000 | 5.000 | 6.000 | 5.000 | 3.000 | 4.000 | 4.000 | 5.000 | 2.000 | 2.000 | 5.000 | 7.000 | 6.000 | 5.000 | 5.000 | 3.833 | 0.317 |
|  | Ne | 2.909 | 2.462 | 3.200 | 2.286 | 1.600 | 3.556 | 1.280 | 2.000 | 1.000 | 2.462 | 4.571 | 5.333 | 4.000 | 2.571 | 3.556 | 3.556 | 4.500 | 1.280 | 1.280 | 4.571 | 6.400 | 4.571 | 4.000 | 4.571 | 3.230 | 0.295 |
|  | Ho | 0.750 | 1.000 | 0.500 | 0.500 | 0.500 | 0.750 | 0.250 | 1.000 | 0.000 | 0.500 | 0.750 | 1.000 | 0.750 | 0.333 | 1.000 | 1.000 | 1.000 | 0.250 | 0.250 | 1.000 | 1.000 | 1.000 | 0.500 | 1.000 | 0.691 | 0.065 |
|  | He | 0.656 | 0.594 | 0.688 | 0.563 | 0.375 | 0.719 | 0.219 | 0.500 | 0.000 | 0.594 | 0.781 | 0.813 | 0.750 | 0.611 | 0.719 | 0.719 | 0.778 | 0.219 | 0.219 | 0.781 | 0.844 | 0.781 | 0.750 | 0.781 | 0.602 | 0.047 |
|  | HWE | ns | ns | ns | ns | ns | ns | ns | ns | ns | ns | ns | ns | ns | ns | ns | ns | ns | ns | ns | ns | ns | ns | ns | ns |  |  |
| Hikurangi Margin | N | 4 | 4 | 4 | 4 | 4 | 4 | 4 | 4 | 4 | 4 | 4 | 4 | 3 | 2 | 4 | 3 | 3 | 3 | 4 | 3 | 3 | 3 | 4 | 2 | 3.542 | 0.134 |
|  | Na | 2.000 | 4.000 | 3.000 | 6.000 | 3.000 | 3.000 | 5.000 | 3.000 | 2.000 | 2.000 | 6.000 | 4.000 | 3.000 | 4.000 | 3.000 | 3.000 | 3.000 | 2.000 | 3.000 | 4.000 | 5.000 | 5.000 | 3.000 | 4.000 | 3.542 | 0.241 |
|  | Ne | 2.000 | 3.200 | 2.462 | 4.571 | 2.909 | 2.133 | 4.000 | 2.462 | 1.280 | 1.280 | 5.333 | 3.200 | 2.571 | 4.000 | 2.462 | 2.571 | 2.000 | 1.385 | 1.684 | 3.600 | 4.500 | 4.500 | 1.684 | 4.000 | 2.908 | 0.239 |
|  | Ho | 1.000 | 1.000 | 1.000 | 1.000 | 0.750 | 0.750 | 0.750 | 1.000 | 0.250 | 0.250 | 0.750 | 1.000 | 0.333 | 1.000 | 1.000 | 0.667 | 0.667 | 0.333 | 0.500 | 1.000 | 1.000 | 1.000 | 0.500 | 1.000 | 0.771 | 0.056 |
|  | He | 0.500 | 0.688 | 0.594 | 0.781 | 0.656 | 0.531 | 0.750 | 0.594 | 0.219 | 0.219 | 0.813 | 0.688 | 0.611 | 0.750 | 0.594 | 0.611 | 0.500 | 0.278 | 0.406 | 0.722 | 0.778 | 0.778 | 0.406 | 0.750 | 0.592 | 0.037 |
|  | HWE | ns | ns | ns | ns | ns | ns | ns | ns | ns | ns | ns | ns | ns | ns | ns | ns | ns | ns | ns | ns | ns | ns | ns | ns |  |  |
| Campbell Plateau | N | 8 | 8 | 8 | 8 | 7 | 7 | 8 | 8 | 8 | 8 | 8 | 8 | 8 | 4 | 8 | 7 | 4 | 7 | 8 | 8 | 8 | 7 | 8 | 8 | 7.458 | 0.233 |
|  | Na | 2.000 | 5.000 | 6.000 | 4.000 | 4.000 | 4.000 | 4.000 | 7.000 | 2.000 | 5.000 | 6.000 | 8.000 | 7.000 | 7.000 | 6.000 | 8.000 | 5.000 | 5.000 | 5.000 | 6.000 | 10.000 | 8.000 | 7.000 | 9.000 | 5.833 | 0.411 |
|  | Ne | 1.969 | 3.556 | 2.723 | 2.246 | 3.630 | 2.882 | 1.488 | 5.120 | 1.133 | 2.000 | 3.200 | 5.818 | 4.923 | 6.400 | 3.282 | 6.125 | 4.000 | 2.227 | 2.783 | 3.368 | 8.000 | 7.000 | 4.741 | 6.737 | 3.973 | 0.390 |
|  | Ho | 0.875 | 0.875 | 0.750 | 0.375 | 0.857 | 0.857 | 0.250 | 0.875 | 0.125 | 0.375 | 0.750 | 0.750 | 0.875 | 0.750 | 1.000 | 1.000 | 0.500 | 0.571 | 0.875 | 1.000 | 0.875 | 1.000 | 0.625 | 0.875 | 0.736 | 0.051 |
|  | He | 0.492 | 0.719 | 0.633 | 0.555 | 0.724 | 0.653 | 0.328 | 0.805 | 0.117 | 0.500 | 0.688 | 0.828 | 0.797 | 0.844 | 0.695 | 0.837 | 0.750 | 0.551 | 0.641 | 0.703 | 0.875 | 0.857 | 0.789 | 0.852 | 0.676 | 0.037 |
|  | HWE | ns | ns | ns | ns | ns | ns | ns | ns | ns | ns | ns | ns | ns | ns | ns | ns | ns | ns | ns | ns | ns | ns | ns | ns |  |  |

N = number of individuals successfully amplified, Na = number of different alleles, Ne = number of effective alleles, Ho = observed heterozygosity, He = expected heterozygosity, HWE = test of deviation from Hardy–Weinberg equilibrium. The geomorphic feature groups are only included in this table when the number of individuals successfully amplified was larger than 4.

Significant values of p >0.05 are marked as ns, p < 0.05 are marked as *, p < 0.01 are marked as **, and p < 0.001 are marked as ***. Mono means the locus is monomorphic.

Table S6a. Pairwise F_ST_ values for all (below diagonal) and neutral (above diagonal) loci amongst Goniocorella dumosa populations

|  | NE continental slope | Challenger Plateau | Chatham Rise | Kermadec Ridge |
| --- | --- | --- | --- | --- |
| NE continental slope |  | -0.018 | 0.033 | 0.009 |
| Challenger Plateau | -0.047 |  | -0.006 | 0.015 |
| Chatham Rise | 0.001 | -0.014 |  | 0.044* |
| Kermadec Ridge | -0.012 | 0.003 | 0.024* |  |

Table S6b. Pairwise F_ST_ values for all (below diagonal) and neutral (above diagonal) loci amongst Solenosmilia variabilis populations

|  | Bounty Plateau | Bounty Trough | Campbell Plateau | Chatham Rise | Hikurangi Margin | Kermadec Ridge | Louisville Seamount Chain | Macquarie Ridge |
| --- | --- | --- | --- | --- | --- | --- | --- | --- |
| Bounty Plateau |  | -0.022 | -0.002 | 0.031* | 0.053 | 0.048* | 0.053 | 0.019 |
| Bounty Trough | -0.037 |  | -0.001 | 0.008 | -0.038 | -0.018 | 0.034 | 0.02 |
| Campbell Plateau | 0.02 | -0.003 |  | 0.061** | 0.034 | 0.086** | 0.115** | 0.061** |
| Chatham Rise | 0.025 | 0.003 | 0.048** |  | 0.033* | 0.022** | 0.043** | -0.009 |
| Hikurangi Margin | 0.094 | 0.02 | 0.052 | 0.048** |  | -0.008 | 0.068* | 0.038* |
| Kermadec Ridge | 0.035 | -0.014 | 0.061** | 0.014** | 0.008 |  | 0.032** | 0.031** |
| Louisville Seamount Chain | 0.06 | 0.029 | 0.085** | 0.035** | 0.081** | 0.027** |  | 0.045** |
| Macquarie Ridge | 0.027 | 0.013 | 0.041** | -0.008 | 0.030* | 0.018** | 0.036** |  |

Significant values of p < 0.05 are marked as *, and p < 0.01 are marked as **.

Data for *M. oculata* not shown because all *F_ST_* values were not statistically significant.

Table S7. Ne Estimator results for effective population size (*Ne*) at different spatial scales

| Species | Geographic scale | Loci data set | Population | N | P_critical_ (1/2N) | *N*e | Lower CI | Upper CI |
| --- | --- | --- | --- | --- | --- | --- | --- | --- |
| *Goniocorella dumosa* | North-Central-South | All | North | 27 | 0.019 | 56.6 | 40.5 | 90.0 |
|  |  | All | Central | 77 | 0.007 | 16.9 | 15.5 | 18.5 |
|  |  | All | South | 4 | 0.125 | ∞ | ∞ | ∞ |
|  |  | Neutral | North | 27 | 0.019 | 66.5 | 32.5 | 699.3 |
|  |  | Neutral | Central | 77 | 0.007 | 26.1 | 21.6 | 32.2 |
|  |  | Neutral | South | 4 | 0.125 | ∞ | ∞ | ∞ |
|  | Geomorphic features | All | NE continental slope | 4 | 0.125 | ∞ | ∞ | ∞ |
|  |  | All | Challenger Plateau | 7 | 0.071 | ∞ | ∞ | ∞ |
|  |  | All | Chatham Rise | 77 | 0.007 | 26.6 | 24.4 | 29.1 |
|  |  | All | Kermadec Ridge | 12 | 0.042 | 40.3 | 19.5 | 452.8 |
|  |  | Neutral | NE continental slope | 4 | 0.125 | ∞ | ∞ | ∞ |
|  |  | Neutral | Challenger Plateau | 7 | 0.071 | ∞ | 26.6 | ∞ |
|  |  | Neutral | Chatham Rise | 77 | 0.007 | 25.2 | 20.7 | 31.0 |
|  |  | Neutral | Kermadec Ridge | 12 | 0.042 | 29.8 | 9.1 | ∞ |
| *Madrepora oculata* | North-Central-South | All | North | 17 | 0.030 | 58.3 | 17.1 | ∞ |
|  |  | All | Central | 39 | 0.013 | 37.9 | 24.8 | 76.7 |
|  |  | All | South | 14 | 0.036 | ∞ | 46.6 | ∞ |
|  |  | Neutral | North | 19 | 0.026 | 147.4 | 12.6 | ∞ |
|  |  | Neutral | Central | 53 | 0.009 | 32.4 | 16.2 | 159.2 |
|  |  | Neutral | South | 21 | 0.024 | ∞ | 15.7 | ∞ |
| *Solenosmilia variabilis* | Northern-Southern | All | Northern | 169 | 0.003 | 73.0 | 70.6 | 75.6 |
|  |  | All | Southern | 39 | 0.013 | 19.0 | 17.3 | 21.0 |
|  |  | Neutral | Northern | 169 | 0.003 | 70.4 | 66.1 | 75.1 |
|  |  | Neutral | Southern | 39 | 0.013 | 12.1 | 10.4 | 14.0 |
|  | North-Central-South | All | North | 84 | 0.006 | 65.8 | 62.0 | 69.9 |
|  |  | All | Central | 85 | 0.006 | 41.5 | 39.1 | 44.2 |
|  |  | All | South | 39 | 0.013 | 19.0 | 17.3 | 21.0 |
|  |  | Neutral | North | 84 | 0.006 | 62.7 | 56.2 | 70.5 |
|  |  | Neutral | Central | 85 | 0.006 | 39.9 | 36.0 | 44.5 |
|  |  | Neutral | South | 39 | 0.013 | 12.1 | 10.4 | 14.0 |
|  | Geomorphic features | All | Bounty Plateau | 4 | 0.125 | ∞ | ∞ | ∞ |
|  |  | All | Bounty Trough | 5 | 0.100 | ∞ | ∞ | ∞ |
|  |  | All | Cambell Plateau | 8 | 0.063 | 210.6 | 31.0 | ∞ |
|  |  | All | Chatham Rise | 85 | 0.006 | 41.5 | 39.1 | 44.2 |
|  |  | All | Hikurangi Margin | 4 | 0.125 | ∞ | ∞ | ∞ |
|  |  | All | Kermadec Ridge | 41 | 0.012 | 56.0 | 49.2 | 64.8 |
|  |  | All | Louisville Seamount Chain | 38 | 0.013 | 31.4 | 28.4 | 35.1 |
|  |  | All | Macquarie Ridge | 15 | 0.033 | 21.1 | 12.6 | 47.7 |
|  |  | Neutral | Bounty Plateau | 4 | 0.125 | ∞ | ∞ | ∞ |
|  |  | Neutral | Bounty Trough | 5 | 0.100 | ∞ | ∞ | ∞ |
|  |  | Neutral | Cambell Plateau | 8 | 0.063 | 63.4 | 12.4 | ∞ |
|  |  | Neutral | Chatham Rise | 85 | 0.006 | 39.9 | 36.0 | 44.5 |
|  |  | Neutral | Hikurangi Margin | 4 | 0.125 | ∞ | ∞ | ∞ |
|  |  | Neutral | Kermadec Ridge | 41 | 0.012 | 66.3 | 51.1 | 91.5 |
|  |  | Neutral | Louisville Seamount Chain | 38 | 0.013 | 27.8 | 23.4 | 33.6 |
|  |  | Neutral | Macquarie Ridge | 15 | 0.033 | 12.4 | 6.8 | 28.4 |

Table S8a. *Goniocorella dumosa*. Numbers of individuals collected from each sampled site (region) that were assigned to each sampled potential source population. GeneClass2 results for all loci where values are not in brackets an d for reduced locus subset (values in brackets).

|  |  | **Assigned to** | | | Total |
| --- | --- | --- | --- | --- | --- |
|  |  | Centre | North | South |  |
| **Sampled from** | Centre | 57 (45) | 12 (18) | 8 (14) | 77 (77) |
|  | North | 9 (14) | 16 (11) | 2 (2) | 27 (27) |
|  | South | 1 (3) | 1 (1) | 2 (0) | 4 (4) |
| Total | | 67 (62) | 29 (30) | 12 (16) | 108 (108) |

Table S8b. *Goniocorella dumosa*. Numbers of first generation migrants from each sampled site (region) that were assigned to each sampled potential source population. GeneClass2 results for all loci where values are not in brackets and for reduced locus subset (values in brackets).

|  | | **Assigned to** | | | Total |
| --- | --- | --- | --- | --- | --- |
|  |  | Centre | North | South |  |
| **Sampled from** | Centre | - | 12 (18) | 8 (14) | 20 (32) |
|  | North | 9 (14) | - | 2 (2) | 11 (16) |
|  | South | 1 (3) | 1 (1) | - | 2 (4) |
| Total | | 10 (17) | 13 (19) | 10 (16) | 33 (52) |

Table S8c. *Goniocorella dumosa*. Numbers of individuals collected from each sampled site (geomorphic feature) that were assigned to each sampled potential source population. GeneClass2 results for all loci where values are not in brackets and for reduced locus subset (values in brackets).

|  | | **Assigned to** | | | | Total |
| --- | --- | --- | --- | --- | --- | --- |
|  |  | NE continental slope | Challenger Plateau | Chatham Rise | Kermadec Ridge |  |
| **Sampled from** | NE continental slope | 3 (1) | 0 (0) | 1 (1) | 0 (2) | 4 (4) |
|  | Challenger Plateau | 0 (0) | 1 (1) | 4 (4) | 2 (2) | 7 (7) |
|  | Chatham Rise | 6 (6) | 5 (10) | 56 (50) | 10 (11) | 77 (77) |
|  | Kermadec Ridge | 0 (1) | 3 (2) | 4 (6) | 5 (3) | 12 (12) |
| Total | | 9 (8) | 9 (13) | 65 (61) | 17 (18) | 100 (100) |

Table S8d. *Goniocorella dumosa*. Numbers of first generation migrants from each sampled site (geomorphic feature) that were assigned to each sampled potential source population. GeneClass2 results for all loci where values are not in brackets and for reduced locus subset (values in brackets).

|  | | **Assigned to** | | | | Total |
| --- | --- | --- | --- | --- | --- | --- |
|  |  | NE continental slope | Challenger Plateau | Chatham Rise | Kermadec Ridge |  |
| **Sampled from** | NE continental slope | - | 0 (0) | 1 (1) | 0 (2) | 1 (3) |
|  | Challenger Plateau | 0 (0) | - | 4 (4) | 2 (2) | 6 (6) |
|  | Chatham Rise | 6 (4) | 5 (12) | - | 10 (11) | 21 (27) |
|  | Kermadec Ridge | 0 (1) | 3 (2) | 4 (6) | - | 7 (9) |
| Total | | 6 (5) | 8 (14) | 9 (11) | 12 (15) | 35 (45) |

Table S8e. *Madrepora oculata*. Numbers of individuals collected from each sampled site (region) that were assigned to each sampled potential source population. GeneClass2 results for all loci where values are not in brackets and for reduced locus subset (values in brackets).

|  | | **Assigned to** | | | Total |
| --- | --- | --- | --- | --- | --- |
|  |  | Centre | North | South |  |
| **Sampled from** | Centre | 15 (20) | 8 (13) | 16 (16) | 39 (49) |
|  | North | 8 (9) | 6 (6) | 3 (4) | 17 (19) |
|  | South | 11 (7) | 0 (2) | 3 (9) | 14 (18) |
| Total | | 34 (36) | 14 (21) | 22 (24) | 70 (86) |

Table S8f. *Madrepora oculata*. Numbers of first generation migrants from each sampled site (region) that were assigned to each sampled potential source population. GeneClass2 results for all loci where values are not in brackets and for reduced locus subset (values in brackets).

|  | | **Assigned to** | | | Total |
| --- | --- | --- | --- | --- | --- |
|  |  | Centre | North | South |  |
| **Sampled from** | Centre | - | 8 (13) | 16 (16) | 24 (29) |
|  | North | 8 (9) | - | 3 (5) | 11 (14) |
|  | South | 11 (7) | 0 (2) | - | 11 (9) |
| Total | | 19 (16) | 8 (15) | 19 (21) | 46 (52) |

Table S8g. *Solenosmilia variabilis*. Numbers of individuals collected from each sampled site (region) that were assigned to each sampled potential source population. GeneClass2 results for all loci where values are not in brackets and for reduced locus subset (values in brackets).

|  | | **Assigned to** | | Total |
| --- | --- | --- | --- | --- |
|  |  | North | South |  |
| **Sampled from** | North | 126 (117) | 43 (52) | 169 (169) |
|  | South | 24 (23) | 15 (16) | 39 (39) |
| Total | | 150 (140) | 58 (68) | 208 (208) |

Table S8h. *Solenosmilia variabilis*. Numbers of first generation migrants from each sampled site (region) that were assigned to each sampled potential source population. GeneClass2 results for all loci where values are not in brackets and for reduced locus subset (values in brackets).

|  | | **Assigned to** | | Total |
| --- | --- | --- | --- | --- |
|  |  | North | South |  |
| **Sampled from** | North | - | 43 (52) | 43 (52) |
|  | South | 24 (23) | - | 24 (23) |
| Total | | 24 (23) | 43 (52) | 67 (75) |

Table S8i. *Solenosmilia variabilis*. Numbers of individuals collected from each sampled site (region) that were assigned to each sampled potential source population. GeneClass2 results for all loci where values are not in brackets and for reduced locus subset (values in brackets).

|  | | **Assigned to** | | | Total |
| --- | --- | --- | --- | --- | --- |
|  |  | North | Centre | South |  |
| **Sampled from** | North | 45 (47) | 29 (22) | 10 (15) | 85 (84) |
|  | Centre | 19 (20) | 36 (39) | 30 (26) | 85 (85) |
|  | South | 7 (9) | 20 (15) | 12 (15) | 39 (39) |
| Total | | 71 (76) | 86 (76) | 52 (56) | 208 (208) |

Table S8j. *Solenosmilia variabilis*. Numbers of first generation migrants from each sampled site (region) that were assigned to each sampled potential source population. GeneClass2 results for all loci where values are not in brackets and for reduced locus subset (values in brackets).

|  | | **Assigned to** | | | Total |
| --- | --- | --- | --- | --- | --- |
|  |  | North | Centre | South |  |
| **Sampled from** | North | - | 29 (22) | 10 (15) | 39 (37) |
|  | Centre | 19 (20) | - | 29 (26) | 48 (46) |
|  | South | 7 (9) | 20 (15) | - | 27 (24) |
| Total | | 26 (29) | 49 (37) | 39 (41) | 115 (107) |

Table S8k. *Solenosmilia variabilis*. Numbers of individuals collected from each sampled site (geomorphic feature) that were assigned to each sampled potential source population. GeneClass2 results for all loci where values are not in brackets and for reduced locus subset (values in brackets).

|  | | **Assigned to** | | | | | | | | Total |
| --- | --- | --- | --- | --- | --- | --- | --- | --- | --- | --- |
|  |  | Bounty Plateau | Bounty Trough | Campbell Plateau | Chatham Rise | Hikurangi Margin | Kermadec Ridge | Louisville Seamount Chain | Macquarie Ridge |  |
| **Sampled from** | Bounty Plateau | 1 (0) | 0 (1) | 1 (0) | 1 (3) | 0 (0) | 1 (0) | 0 (0) | 0 (0) | 4 (4) |
|  | Bounty Trough | 0 (0) | 0 (1) | 0 (0) | 4 (1) | 0 (1) | 0 (1) | 1 (1) | 0 (0) | 5 (5) |
|  | Campbell Plateau | 1 (1) | 0 (1) | 0 (1) | 4 (2) | 0 (0) | 3 (3) | 0 (0) | 0 (0) | 8 (8) |
|  | Chatham Rise | 0 (5) | 6 (6) | 3 (3) | 36 (32) | 3 (6) | 15 (12) | 5 (5) | 17 (16) | 85 (85) |
|  | Hikurangi Margin | 0 (0) | 0 (1) | 0 (1) | 2 (0) | 0 (0) | 2 (2) | 0 (0) | 0 (0) | 4 (4) |
|  | Kermadec Ridge | 0 (1) | 2 (5) | 1 (1) | 15 (10) | 1 (1) | 16 (18) | 3 (4) | 3 (1) | 41 (41) |
|  | Louisville Seamount Chain | 0 (0) | 0 (0) | 0 (0) | 6 (3) | 0 (0) | 2 (3) | 26 (27) | 4 (5) | 38 (38) |
|  | Macquarie Ridge | 0 (1) | 0 (0) | 0 (0) | 6 (5) | 0 (1) | 3 (1) | 1 (1) | 5 (6) | 15 (15) |
|  | Total | 2 (8) | 8 (15) | 5 (6) | 74 (56) | 4 (9) | 42 (40) | 36 (38) | 29 (28) | 200 (200) |

Table S8l. *Solenosmilia variabilis*. Numbers of first generation migrants from each sampled site (geomorphic feature) that were assigned to each sampled potential source population. GeneClass2 results for all loci where values are not in brackets and for reduced locus subset (values in brackets).

|  | | **Assigned to** | | | | | | | | Total |
| --- | --- | --- | --- | --- | --- | --- | --- | --- | --- | --- |
|  |  | Bounty Plateau | Bounty Trough | Campbell Plateau | Chatham Rise | Hikurangi Margin | Kermadec Ridge | Louisville Seamount Chain | Macquarie Ridge |  |
| **Sampled from** | Bounty Plateau | - | 0 (1) | 1 (0) | 1 (3) | 0 (0) | 1 (0) | 0 (0) | 0 (0) | 3 (4) |
|  | Bounty Trough | 0 (0) | - | 0 (0) | 4 (1) | 0 (1) | 0 (1) | 1 (1) | 0 (0) | 5 (4) |
|  | Campbell Plateau | 1 (1) | 0 (1) | - | 4 (2) | 0 (0) | 3 (3) | 0 (0) | 0 (0) | 8 (7) |
|  | Chatham Rise | 0 (5) | 6 (6) | 3 (3) | - | 3 (6) | 15 (12) | 5 (5) | 17 (16) | 49 (53) |
|  | Hikurangi Margin | 0 (0) | 0 (1) | 0 (1) | 2 (0) | - | 2 (2) | 0 (0) | 0 (0) | 4 (4) |
|  | Kermadec Ridge | 0 (1) | 2 (5) | 1 (1) | 15 (10) | 1 (1) | - | 3 (4) | 3 (1) | 25 (23) |
|  | Louisville Seamount Chain | 0 (0) | 0 (0) | 0 (3) | 6 (0) | 0 (0) | 2 (3) | - | 4 (5) | 12 (11) |
|  | Macquarie Ridge | 0 (1) | 0 (0) | 0 (0) | 6 (5) | 0 (1) | 3 (1) | 1 (1) | - | 10 (9) |
| Total | | 1 (8) | 8 (14) | 5 (8) | 38 (21) | 4 (9) | 26 (22) | 10 (11) | 24 (22) | 116 (115) |

Table S9. Information about reproductive life-history characteristics for *Goniocorella dumosa*, *Madrepora oculata* and *Solenosmilia variabilis*

| Species | Mode of Sexual  reproduction | Max Oocyte  diameter | Fecundity  (oocytes per polyp) | Methods | Production | Larvae |
| --- | --- | --- | --- | --- | --- | --- |
| *Goniocorella dumosa* | Gonochoric (1) | 135 µm (1) | 480 (1) | Spawner (1) | Seasonal (1) | - |
| *Madrepora oculata* | Functional Gonochoric (1,2,3) | 350 µm (1)  650 µm (2) | 10 (1) | Broadcast Spawner (1,2) | Periodic (1) /Continuous (2) | Lecithotrophic (1,2) |
| *Solenosmilia variabilis* | Gonochoric (1,2) | 165 µm (1) 337µm (2) | 290 (1) | Broadcast Spawner (1,2) | Seasonal (1) / Continuous (2, 4) | Lecithotrophic (1,2) |

1. Data from: Waller, R. G., & Tyler, P. A. (2005). The reproductive biology of two deep-water, reef-building scleractinians from the NE Atlantic Ocean. *Coral Reefs*, 24(3), 514–522.

2. Data from: Pires, D. O., Silva, J. C., & Bastos, N. D. (2014). Reproduction of deep-sea reef-building corals from the southwestern Atlantic. *Deep-Sea Research Part II: Topical Studies in Oceanography*, 99, 51–63.

3. A few hermaphroditic colonies were observed.

4. A reproductive peak between April and September (Autumn–Spring)


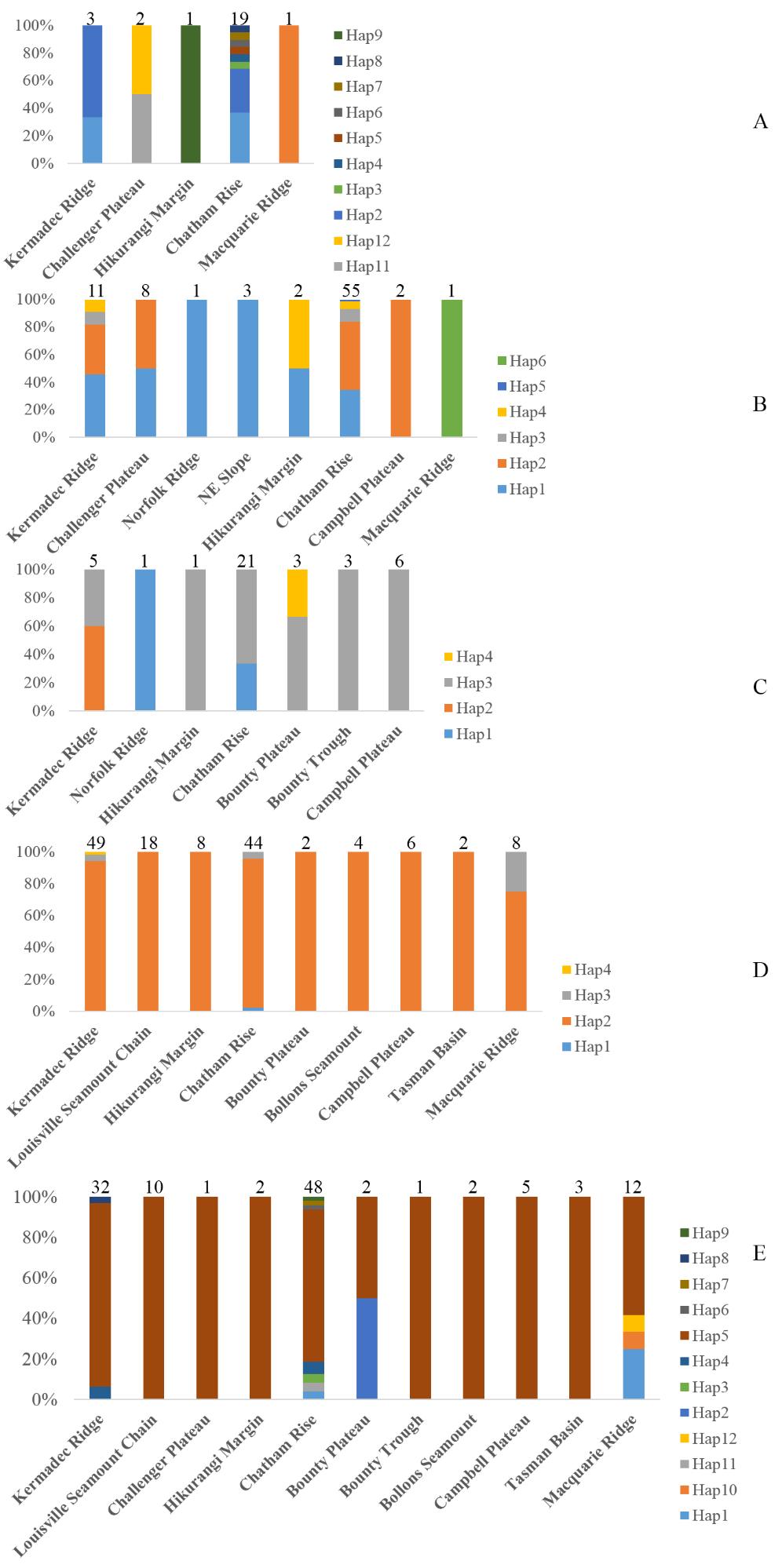


Figure S1. Haplotype distributions for *Goniocorella dumosa* *D-loop* (A) *ITS* (B), *Madrepora oculata ITS* (C), *Solenosmilia variabilis D-loop* (D) and *ITS* (E) across populations on geomorphic features. The numbers at the top of each bar are sample sizes.


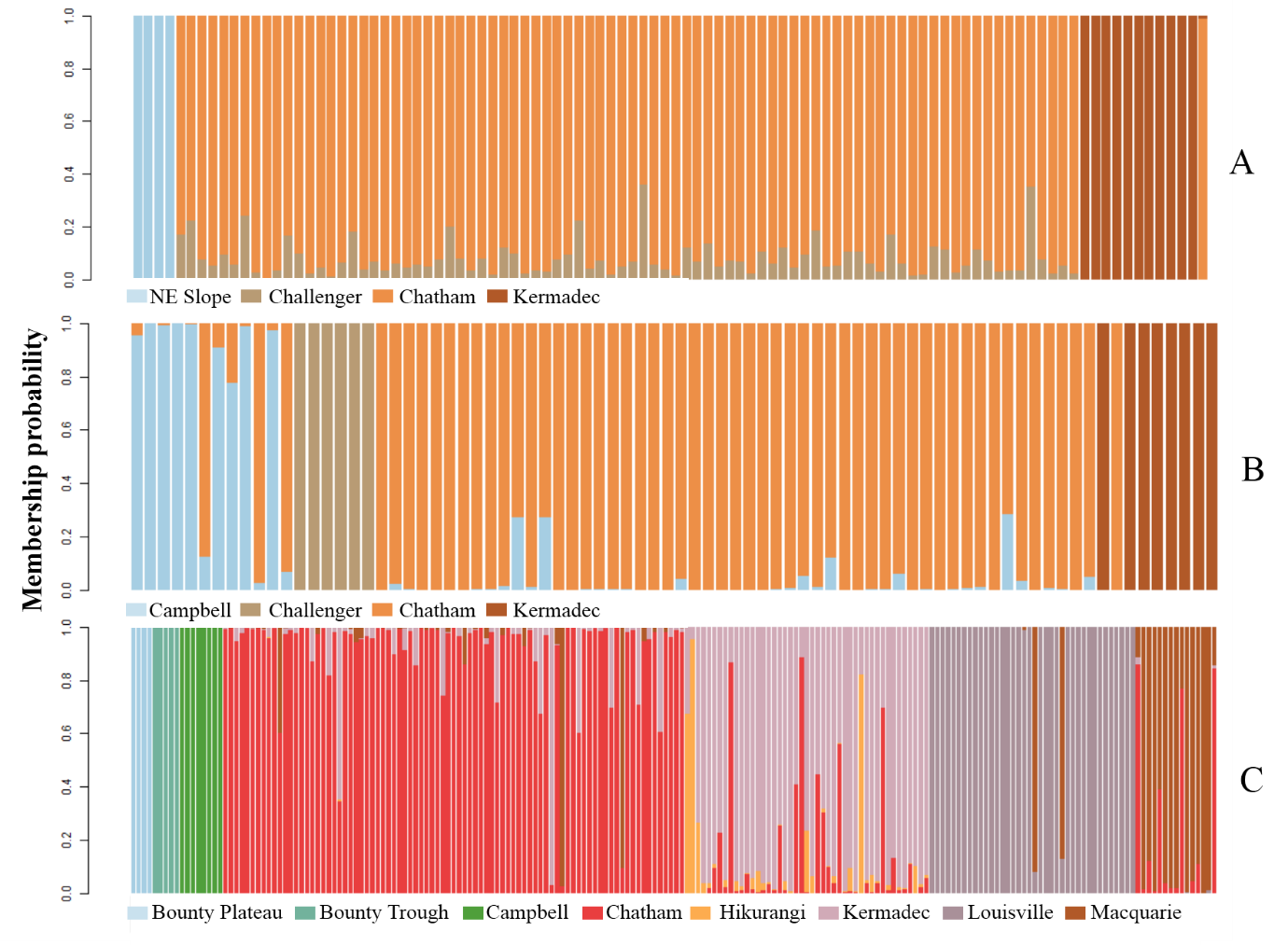


Figure S2. Posterior probability membership complots from the DAPC for *Goniocorella dumosa* (A), *Madrepora oculata* (B) and *Solenosmilia variabilis* (C)


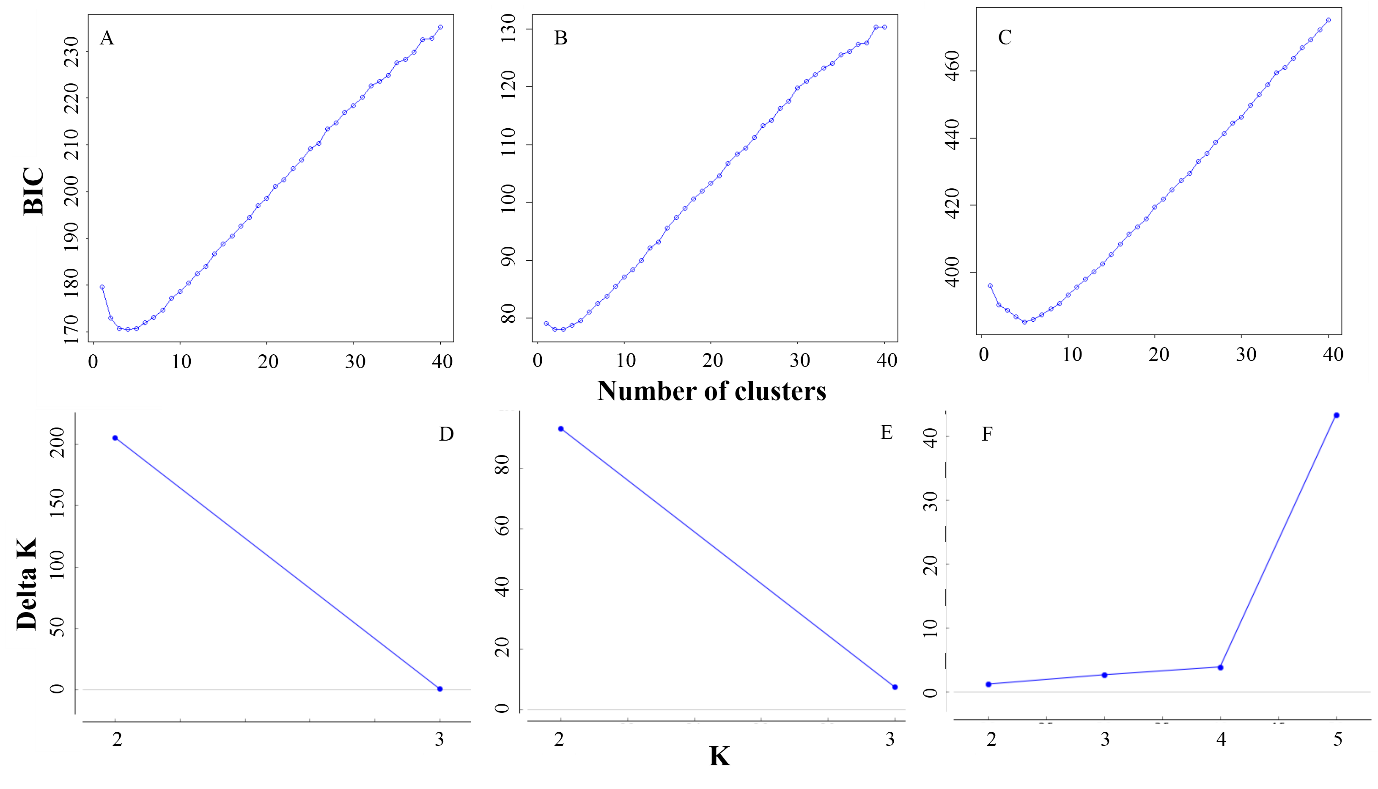


Figure S3. Optimal genetic cluster numbers predicted by BIC for *Goniocorella dumosa* (A), *Madrepora oculata* (B) and *Solenosmilia variabilis* (C) and by Structure Harvester for *Goniocorella dumosa* (D), *Madrepora oculata* (E) and *Solenosmilia variabilis* (F)


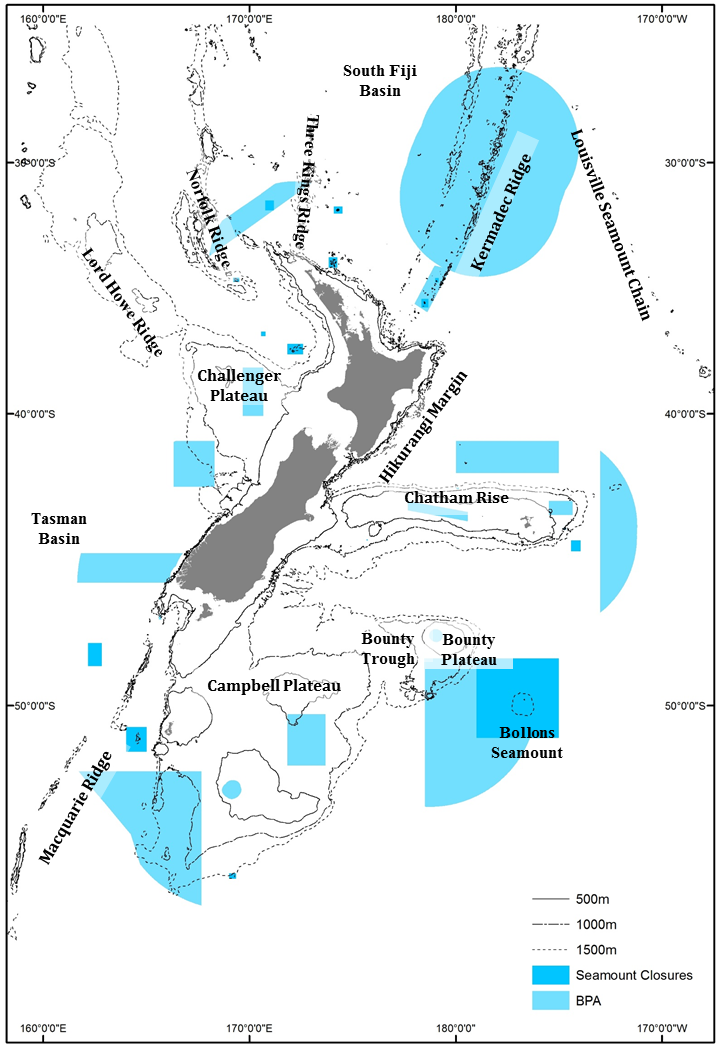


Figure S4. Existing protected offshore areas within the New Zealand EEZ.

BPA = benthic protection area

**Acknowledgements**

This work was funded by the New Zealand Ministry of Business, Innovation and Employment as part of the NIWA-led project ‘‘Predicting the occurrence of vulnerable marine ecosystems for planning spatial management in the South Pacific region’’ (CO1X1229). The research was also benefitted from additional funding provided by NIWA under the Marine Biological Resources programme (Objective 1: Discovery and definition of the marine biota of New Zealand). Specimens and data were provided by the NIWA Invertebrate Collection including material from the following projects: “Seamounts: their importance to fisheries and marine ecosystems”, undertaken by the NIWA and funded by the former New Zealand Foundation for Research, Science and Technology (FRST) with additional funding from the former Ministry of Fisheries (MFish), NOAA Satellite Operations Facility (TAN0413) and the Census of Marine Life field programme on seamounts, CenSeam (TAN0604); RENEWZ I (TAN0616) voyage, were components of the project ‘Exploration of Chemosynthetic Habitats of the New Zealand Region’, funded by NOAA Ocean Exploration and NIWA, with co-funding from Woods Hole Oceanographic Institution (WHOI), Scripps Oceanographic Institution, and the University of Hawaii; Ocean Survey 20/20 Chatham/Challenger Biodiversity and Seabed Habitat Project, jointly funded by MFish, Land Information New Zealand (LINZ), NIWA, and Department of Conservation; New Zealand-Australian “MacRidge 2” research voyage (TAN0803), the biological component of which was part of NIWA’s research project “Seamounts: their importance to fisheries and marine ecosystems” funded by FRST, and CSIRO’s Division of Marine and Atmospheric Research project “Biodiversity Voyages of Discovery” funded by the CSIRO Wealth from Oceans Flagship; ‘Impact of resource use on vulnerable deep-sea communities’ (DSCA: TAN1004, TAN1206, TAN1503), funded by the Ministry of Business, Innovation & Employment (MBIE); Kermadec Arc Minerals (KARMA) voyage, funded by MBIE, in collaboration with Auckland University, GNS Science, and WHOI; Ocean Survey 20/20 Mapping the Mineral Resources of the Kermadec Arc Project, funded by LINZ, Institute of Geological and Nuclear Science, NIWA, and WHOI; Biogenic Habitats on the Continental Shelf project (voyages TAN1105), funded by MPI, FRST, NIWA and Oceans Survey 20/20 ship time (LINZ); Nascent Inter-Ridge Volcanic And Neotectonic Activity (NIRVANA, TAN1213) voyage, funded by the Ministry for Primary Industries (MPI), in collaboration with Auckland University, GNS, and the University of New Hampshire (USA) and with funding from the DSCA project, funded by MBIE; NIWA/GNS Oceans Survey 20/20 Reinga voyage (TAN1312), funded by LINZ and New Zealand Petroleum & Minerals; South Pacific Vulnerable Marine Ecosystems Project (C01X1229) funded by MBIE; Scientific Observer Program funded by MFish/MPI; NIWA trawl surveys funded by MFish/MPI; various campaigns and cruises of the New Zealand Oceanographic Institution.
